# Supplementary material for: Integrated Management of Childhood Illnesses (IMCI): a mixed-methods study on implementation, knowledge and resource availability in Malawi
Source: BMJ Paediatr Open. 2021 Apr 30;5(1):e001044. doi: 10.1136/bmjpo-2021-001044 (PMC8098945; doi:10.1136/bmjpo-2021-001044)
Supplement: Supplementary data [file bmjpo-2021-001044supp001.pdf]

Appendix 1: Summary of healthcare worker cadres and their role

Qualification	Definition
Health Surveillance Assistants	Community level workers with 6 weeks of initial health pre-service training. Generally, provide health promotion and preventive health care through door to door or outreach clinics. Diagnose and treat some illness as part of iCCM in more remote/underserviced areas [1].
Hospital Attendant	Workers who perform routine patient personal care as directed by more senior health personnel. It includes orderlies and nursing aides [2].
Medical Assistants	Two years of clinical training [1] to gain a certificate in clinical medicine. Perform clinical duties (except for surgical procedures) [3].
Clinical Officer	Mid-level practitioners who receive 3-4 years of training and often manage health facilities and fill the role of doctors due to workforce shortages [1, 4]

1. Makwero M. Delivery of primary health care in Malawi. *African Journal of Primary Health Care & Family Medicine*. 2018;10(1)
2. Malawi Health Workforce Observatory. *Human Resources for Health Country Profile Malawi,*. 2010.
3. Muula A. Case for Clinical Officers and Medical Assistants in Malawi. *Croatian Medical Journal*. 2009;50(1):77-78.
4. Jiskoot P. On-the-job training of clinical officers in Malawi. *Malawi Medical Journal*. 2008;20(3):74-77.

Appendix 2: Healthcare worker focus group discussion topic guide

1. Current IMCI

- When did you last have IMCI training? Can you describe the training?
- Can you describe what IMCI is?
- Do you think you implement IMCI in your setting?
- Are there any barriers you face in implementing IMCI?
- Are there any benefits you think of implementing IMCI?

2. Current emergency treatment

- Can you give a recent example of child you saw, that you would describe as an emergency?
  - Probe: do others recognise this example, ask for multiple different ones, you can give an example
- Can you describe what you did with this child?
  - Probe: would everyone do the same, what would they do different
- What would you have liked to do in this situation?
- How did you feel about your management of this case?
- How often do you see children like this?
- Do you use any tools or algorithms (guidelines) when assessing emergency cases?
- When managing emergency cases, how do you work? Is it alone, or in a team? Who is part of the team?

3. Current referral procedure

- How often do you refer a child to the hospital? For example, this week how many children has everyone referred?
- Can you describe the process of referral?
  - Probe: documentation, explaining to the caregiver, organising transport
- Do all caregivers complete the referral?
- Are there any barriers you face in referring children?
- Are there any benefits to referring children?

4. ETAT awareness

- How do you decide which order to see patients in your clinic?
- Have you heard of ETAT? Can you describe ETAT?
- Have you ever had training in ETAT? What about other types of triaging?

5. ETAT preparedness

- What steps would need to happen for you implement ETAT?
- Do you have the equipment you need? Can you give examples?
- Do you have the staff you need?
- Are there any barriers to implementing ETAT? And barriers to adequate management of severely ill children?
- Are there any benefits to implementing ETAT in your setting?

Kilov K, *et al. BMJ Paediatrics Open* 2021; 5:e001044. doi: 10.1136/bmjpo-2021-001044

Appendix 3: Summary of healthcare worker IMCI knowledge scores, with 10 being the maximum score

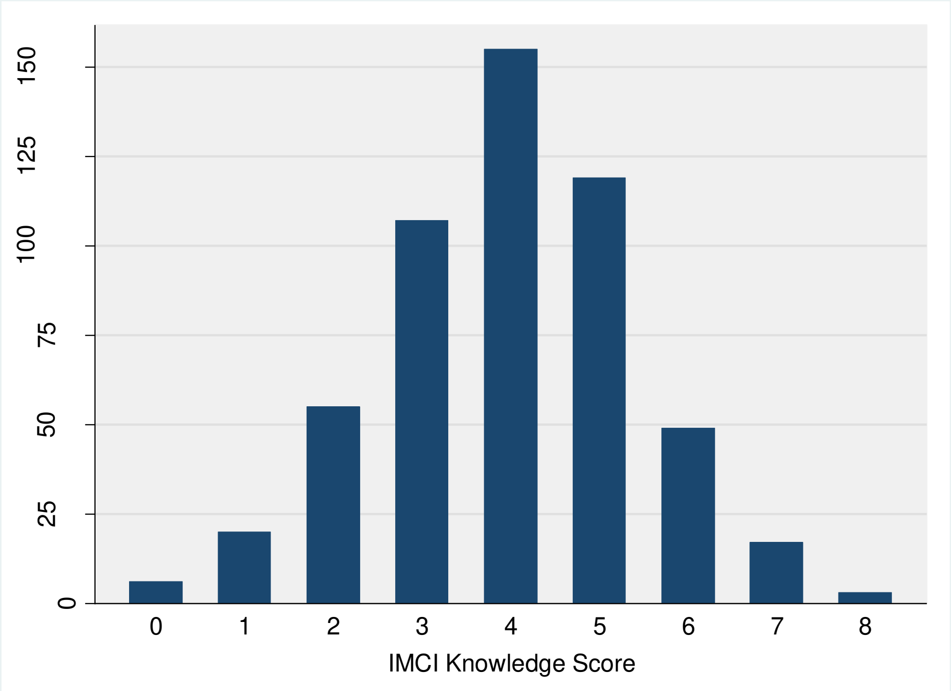

Kilov K, *et al. BMJ Paediatrics Open* 2021; 5:e001044. doi: 10.1136/bmjpo-2021-001044

Appendix 4: Healthcare provider IMCI training and knowledge survey

1.1 Have you ever had IMCI training?	Yes / No	If YES, go to 1.4
1.2 Have you ever been given an orientation or briefing on IMCI?	Yes / No	If NO, go to 1.9
1.3 Who gave you this orientation/ briefing?	1. Facility in-charge<br>2. Other facility staff<br>3. DHMT representative<br>4. Other	Go to 1.9
1.4 When was your first training?	______ year	
1.5 Who provided this first training?	1. MoH/DHMT<br>2. NGO<br>3. Research project<br>4. During school/training<br>5. Other	
1.6 Have you had a refresher training or active mentorship since?	Y / N	If NO, go to 1.9
1.7 When was the refresher?	______ year	
1.8 Who provided this refresher training?	1. MoH / DHTM<br>2. NGO<br>3. Research project<br>4. During school/training<br>5. Other	Select all that apply
1.9 What is a child's classification if he is 10 months old, has had a cough that lasted two days, has a breathing rate of 46 breaths per minute and chest indrawing?[1]	1. Cough or cold<br>2. Pneumonia<br>3. Severe pneumonia<br>4. Very severe febrile disease<br>5. Don't know	Select one
1.10 What are the four main symptoms for which every sick child should be checked?	1. Malnutrition, cough, vitamin A, ear problems<br>2. Anemia, fever, diarrhea, ear problem<br>3. Cough, diarrhea, malnutrition, ear problem<br>4. Cough, diarrhea, fever, ear problem<br>5. Don't know	Select the best answer
1.11 For each of the following cases, select **Yes** if urgent referral is needed or select **No** if urgent referral is not needed.		
1.11a A 6-month-old boy does not have general danger signs. He is classified with: MASTOIDITIS and NO ANEMIA AND NOT VERY LOW WEIGHT	1. Yes<br>2. No<br>3. Don't know	
1.11b A 7-month-old girl does not have general danger signs. She is classified with: COUGH and DIARRHEA	1. Yes<br>2. No<br>3. Don't know	

4

1.11c A 9-month-old boy is lethargic. He is classified with: DIARRHEA and WITH SEVERE DEHYDRATION	1. Yes<br>2. No<br>3. Don't know	
1.11d A 2-year-old girl does not have general danger signs. She is classified with: DIARRHEA and SEVERE DEHYDRATION and SEVERE MALNUTRITION	1. Yes<br>2. No<br>3. Don't know	
1.12 If a child has any of the four general danger signs, you should urgently refer him to hospital for treatment, These signs are:	1. Unable to drink/feed<br>2. Severe cough<br>3. Convulsions<br>4. Vomiting everything<br>5. Lethargy / unconsciousness<br>6. Bloody stools<br>7. Don't know	Select four from the list
1.13 According to IMCI, a mother of a sick child should be counselled about what topics:	1. Importance of fluids and feeding<br>2. When to return to the clinic immediately<br>3. Her own health<br>4. Immunisation<br>5. When to return for a follow-up visit<br>6. The treatments being given to the child<br>7. Family planning<br>8. Don't know	Select all that apply
1.14 What are two signs that are used to classify severe malnutrition	1. Small arm circumference<br>2. Visible severe wasting<br>3. Oedema of both feet<br>4. Severe dehydration<br>5. Don't know	Select two from the list
1.15 To classify the dehydration status of a child with diarrhoea you will look:	1. At the general condition<br>2. For sunken eyes<br>3. For oedema of both feet<br>4. If the child is drinking eagerly<br>5. For palmar pallor<br>6. For a swollen abdomen<br>7. Don't know	Select all that apply
