# Supplementary material for: Integrated Management of Childhood Illnesses (IMCI): a mixed-methods study on implementation, knowledge and resource availability in Malawi
Source: BMJ Paediatr Open. 2021 Apr 30;5(1):e001044. doi: 10.1136/bmjpo-2021-001044 (PMC8098945; doi:10.1136/bmjpo-2021-001044)
Supplement: Author's manuscript [file bmjpo-2021-001044.draft_revisions.pdf]

BMJ Paediatrics Open

BMJ Paediatrics Open is committed to open peer review. As part of this commitment we make the peer review history of every article we publish publicly available.

When an article is published we post the peer reviewers' comments and the authors' responses online. We also post the versions of the paper that were used during peer review. These are the versions that the peer review comments apply to.

The versions of the paper that follow are the versions that were submitted during the peer review process. They are not the versions of record or the final published versions. They should not be cited or distributed as the published version of this manuscript.

BMJ Paediatrics Open is an open access journal and the full, final, typeset and author-corrected version of record of the manuscript is available on our site with no access controls, subscription charges or pay-per-view fees (http://bmjpaedsopen.bmj.com).

If you have any questions on BMJ Paediatrics Open's open peer review process please email info.bmjpo@bmj.com

BMJ Paediatrics Open

Integrated Management of Childhood Illnesses (IMCI): A mixed-methods study on implementation, knowledge and resource availability in Malawi

Journal:	*BMJ Paediatrics Open*
Manuscript ID	bmjpo-2021-001044
Article Type:	Original research
Date Submitted by the Author:	05-Feb-2021
Complete List of Authors:	Kilov, Kim; Karolinska Institute, Department of Global Public Health<br>Hildenwall, Helena; Karolinska Institute, Department of Global Public Health<br>Dube, Albert; Malawi Epidemiology and Intervention Research Unit (MEIRU)<br>Zadutsa, Beatiwel; Parent and Child Health Initiative<br>Banda, Lumbani; Parent and Child Health Initiative<br>Langton, Josephine; University of Malawi College of Medicine, Paediatrics<br>Desmond, Nicola; Malawi-Liverpool-Wellcome Trust Programme<br>Lufesi, Norman; Ministry of Health<br>Makwenda, Charles; Parent and Child Health Initiative<br>King, Carina; University College London, Institute for Global Health; Karolinska Institute, Department of Global Public Health
Keywords:	Health services research

SCHOLARONE™
Manuscripts

I, the Submitting Author has the right to grant and does grant on behalf of all authors of the Work (as defined in the below author licence), an exclusive licence and/or a non-exclusive licence for contributions from authors who are: i) UK Crown employees; ii) where BMJ has agreed a CC-BY licence shall apply, and/or iii) in accordance with the terms applicable for US Federal Government officers or employees acting as part of their official duties; on a worldwide, perpetual, irrevocable, royalty-free basis to BMJ Publishing Group Ltd ("BMJ") its licensees and where the relevant Journal is co-owned by BMJ to the co-owners of the Journal, to publish the Work in this journal and any other BMJ products and to exploit all rights, as set out in our licence.

The Submitting Author accepts and understands that any supply made under these terms is made by BMJ to the Submitting Author unless you are acting as an employee on behalf of your employer or a postgraduate student of an affiliated institution which is paying any applicable article publishing charge ("APC") for Open Access articles. Where the Submitting Author wishes to make the Work available on an Open Access basis (and intends to pay the relevant APC), the terms of reuse of such Open Access shall be governed by a Creative Commons licence – details of these licences and which Creative Commons licence will apply to this Work are set out in our licence referred to above.

Other than as permitted in any relevant BMJ Author's Self Archiving Policies, I confirm this Work has not been accepted for publication elsewhere, is not being considered for publication elsewhere and does not duplicate material already published. I confirm all authors consent to publication of this Work and authorise the granting of this licence.

Confidential: For Review Only

Title: Integrated Management of Childhood Illnesses (IMCI): A mixed-methods study on implementation, knowledge and resource availability in Malawi

Authors: Kim Kilov[1], Helena Hildenwall[1,2,3], Albert Dube[4], Beatiwel Zadutsa[5], Lumbani Banda[5], Josephine Langton[6], Nicola Desmond[7], Norman Lufesi[8], Charles Makwenda[5], Carina King[1,9]*

Affiliations:

1. Department of Global Public Health, Karolinksa Institutet, Stockholm, Sweden
2. Astrid Lindgren Children's Hospital, Karolinska University Hospital, Stockholm, Sweden
3. Department of Clinical Science, Intervention and Technology, Karolinska Institutet, Stockholm, Sweden
4. Malawi Epidemiology and Intervention Research Unit (MEIRU), Lilongwe, Malawi
5. Parent and Child Health Initiative, Lilongwe, Malawi
6. Department of Paediatrics, College of Medicine, Blantyre, Malawi
7. Social Science Unit, Malawi-Liverpool-Wellcome Trust Programme, Blantyre, Malawi
8. Acute Respiratory Infections Unit, Ministry of Health, Lilongwe, Malawi
9. Institute for Global Health, University College London, London, UK

***Corresponding author:**

Department of Global Public Health, Karolinska Institutet, Widerströmska huset, Tomtebodavägen 18B, 171 65 Solna, Sweden. Email: carina.king@ki.se

Keywords:

Integrated Management of Childhood Illness; Malawi; Under-five mortality; Health worker; Knowledge; Resources

Word count: 3644

Confidential: For Review Only

Abstract

Background

The introduction of the World Health Organisation's Integrated Management of Childhood Illnesses guidelines (IMCI) in the mid-1990s contributed to global reductions in under-five mortality. However, issues in quality of care have been reported. We aimed to determine resource availability and healthcare worker knowledge of IMCI guidelines in two Malawian districts.

Methods

We conducted a mixed-method study, including health facility audits, healthcare provider survey and focus group discussions (FGDs) with facility staff. The study was conducted between January to April 2019 in Mchinji (central region) and Zomba (southern region) districts. Quantitative data was described using proportions and chi2 tests; linear regression was conducted to explore factors associated with IMCI knowledge. Qualitative data was analysed using a pragmatic framework approach. Qualitative and quantitative data were analysed and presented separately.

Results

Forty-seven health facilities and 531 healthcare workers were included. Lumefantrine-Artemether and cotrimoxazole were the most available drugs (98% and 96%); while amoxicillin tablets and salbutamol nebuliser solution were the least available (28% and 36%). Respiratory rate timers were the least available piece of equipment, with only 8 (17%) facilities having a functional device. The mean IMCI knowledge score was 3.96 out of 10, and there was a statistically significant association between knowledge and having received refresher training (coeff: 0.42; 95% CI: 0.01-0.82). Four themes were identified in the FGDs: IMCI implementation and practice, barriers to IMCI, benefits of IMCI and sustainability.

Conclusion

We found key gaps in IMCI implementation, however these were not homogenous across facilities, suggesting opportunities to learn from locally adapted IMCI best practices. Improving on-going mentorship, training and supervision should be explored to improve quality of care, and programming which moves away from vertical financing with short term support, to a more holistic approach with embedded sustainability may address the balance of resources for different conditions.

Confidential: For Review Only

Summary Box:

What is known about the subject

- WHO's integrated management of childhood illness guidelines have been important in reducing child deaths in low-resource settings
- However, quality of care issues in IMCI implementation have been noted, including lack of resources, drugs and trained staff
- Training in IMCI is variable, and recommendations for and evidence on the impact of refresher trainings is limited

What this study adds

- In two Malawian district, we found key IMCI implementation gaps; these were not homogenous across facilities suggesting opportunities to learn from locally adapted best practice
- Training alone was not independently associated with improved IMCI knowledge, but having had refresher training was.
- Resource gaps were more prominent for respiratory and diarrhoeal case management than malaria, highlighting issues in vertical financing

Confidential: For Review Only

Introduction

Despite a reduction in under five deaths globally from 12.5 million in 1990 to 5.3 million in 2018, progress has been uneven. The highest burden is seen in Sub-Saharan Africa, with considerable national and sub-national variation (1). A key strategy in the success to date was the introduction of the WHOs Integrated Management of Childhood Illness (IMCI) guidelines (2). At the time, the major causes of child mortality were pneumonia, malaria, measles, malnutrition and diarrhoea (2). More recently this has shifted to neonatal complications (1), while pneumonia remains the leading infectious cause of mortality in children under-five (3).

IMCI was intended for countries with under-five mortality rates higher than 40 per 1000 live births, and focuses on three components: improving case management skills, strengthening health systems and improving community health practices (4, 5). While IMCI has been partially or fully adopted by 100 countries (6, 7), a survey from 2016 reported only 44 countries were considered to be fully implementing (2). Further, IMCI coverage was lowest in countries with the highest mortality rates; of the 26 countries to achieve the Millennium Development Goal 4 - reducing under-five mortality by two thirds, 20 had fully implemented IMCI (2).

Key strengths of IMCI are the holistic approach, rational use of medications and improved quality and efficiency of health service provision (2, 7). However, implementation barriers and inconsistencies have been reported. For example, the WHO guidance for training contains 7 modules taught over 11 days, but local adaptations have led to considerable differences, with examples of both shorter and distance learning approaches (8, 9). Currently there is a lack of standardised guidance on the frequency, content, pedagogical approach and expected learning outcomes for refresher courses.

Malawi was an early adopter of IMCI, first implementing in 2000 (10), and was one of only 10 low-income countries in Sub-Saharan Africa to achieve MDG4 (11). This success has been attributed to proactive policies, scale-up and introduction of vaccinations, coverage of insecticide treated bed nets, IMCI and integrated community case management (10). However, Malawi is not currently on track to achieve the target set out in Sustainable Development Goal 3.2, and faces considerable challenges with an under-resourced health sector with workforce shortages (12). Previous studies have reported suboptimal IMCI implementation for pneumonia, with issues in clinical assessments, quality of diagnosis and antibiotic prescription (13, 14).

Given changes in causes of under-five mortality, varied successes in IMCI implementation and ongoing challenges of under-resourced health systems, it is important to assess if IMCI is effectively supported and implemented. We aimed to describe current IMCI implementation at primary care in

Confidential: For Review Only

Malawi, and determine whether there were sufficient resources and trained staff available to assess and manage children under-five according to these guidelines.

Methods

We conducted a concurrent mixed-methods study, covering all dispensaries, health centres, rural hospitals and Christian Health Association of Malawi (CHAM) hospitals in Zomba and Mchinji districts. Data was collected between January – April 2019. The study included facility audits to assess resource availability, healthcare provider surveys to assess training and knowledge, and focus groups discussions (FGDs) with healthcare providers and facility managers to explore current implementation. Qualitative and quantitative data were analysed and are presented separately.

Setting

Mchinji district is in Malawi's central region, with an under-five population of approximately 90,000 and an under-five mortality rate of 123/1,000 livebirths in the 2015-16 DHS. Zomba is located in the Southern region, with an under-five population of 120,000 and mortality of 54/1,000 livebirths (15, 16). Zomba has a larger urban population and has historically received both higher per capita domestic and external funding (15, 17), while Mchinji has 84% of the population living rurally (15). The health system is made up of three levels (18). Health centres, dispensaries and rural clinics deliver primary care and are linked to community care via Health Surveillance Assistants (HSA) (19). Secondary care is delivered at district hospitals and regional referral hospitals provide tertiary care. Government care is free, and costs of services delivered by CHAM facilities are subsidised through service level agreements resulting in small out of pocket payments (21).

Sampling

All dispensaries, health centres, rural and CHAM hospitals (i.e., frontline facilities) were eligible to be audited. A convenience sample of staff were recruited for the survey, including staff who interact with paediatric patients and were present at the facility at the time of data collection. Qualitative participants were selected using purposive sampling, aiming to conduct three FGDs in each district. Participants were invited to participate by phone by a member of study staff, with permission from the District Health Management Team and senior HSAs. The groups targeted included: 1. HSAs and attendants at health centres; 2. medical assistants and nurses at health centres; 3. clinical officers and medical assistants at rural hospitals. We invited up to 10 participants per group.

Quantitative Data Collection

Confidential: For Review Only

The audit assessed the number of healthcare workers, availability and functionality of essential IMCI equipment (thermometer, respiratory rate timer, mid-upper arm circumference (MUAC) tape, scale, nebuliser) and availability of IMCI drugs (antibiotics, anti-malarials, oral rehydration solution and salbutamol), and whether they were in date. The structured healthcare worker survey included questions on: cadre, previous IMCI training, refresher courses, years since training and refresher training received and knowledge of the 2014 IMCI guidelines. The knowledge questions were adapted from an IMCI computer-based training course evaluation delivered by USAID in Kenya (20).

Data was collected by trained study staff who visited each facility at a pre-arranged time to conduct the audit and surveys. The study staff consisted of clinical officers and monitoring and evaluation officers. The data was collected with support from facility in-charge, pharmacy and health management information staff and included visual inspection of drug stocks to check quantities and expiry dates, equipment functionality and closed questions. Visual inspection by the study staff member was conducted to reduce potential recall and social desirability biases. Data was entered into android tablets using Open Data Kit Collect, with in-built cleaning and skip-pattern rules to promote data quality. The survey was self-completed on an android tablet for healthcare workers who were familiar with smart phone technology. If unfamiliar, the survey was interviewer administered.

Qualitative Data Collection

FGDs were held at healthcare facilities and led by a male Malawian researcher (AD) with experience of qualitative research, supported by a clinical officer to provide IMCI specific knowledge. Discussions were open to be conducted in Chichewa or English, depending on the content and preference of participants. Interviews and discussions were audio-recorded, transcribed and where necessary translated into English. Participants were reimbursed for their travel expenses and provided with refreshments.

Analysis

The audit data was described using proportions, and compared between facility types and districts. Healthcare worker knowledge was summarised into a score with a maximum of 10 points. Mean scores were compared for the following independent variables: IMCI training received, years since training, refresher training received, years since refresher training, cadre, facility type and district. Multivariable linear regression analysis was conducted to assess the association between IMCI knowledge scores and training. The following confounders were considered: district (given different

Confidential: For Review Only

funding levels), facility type, health worker qualification and refresher course. Quantitative analysis was performed using Stata IC 16.0.

FGDs were analysed using a pragmatic framework approach, with pre-defined themes based on the topic guide (21) (Appendix 1). Emergent themes were coded during the analysis. Discrepancies were agreed through discussion, and the interpretation and conclusions shared and discussed with the wider study team. Coding was done by HH, with a sub-set double coded by CK. These codes were then discussed, refined and organised into themes, which were checked by AD for consistency with his interpretation and the local context.

Patient and Public Involvement

Patients and public were not involved in the design or execution of the study. Prior to starting, the protocol was presented to both District Health Management and District Executive Committees (which include local government and civil society representation) in both Zomba and Mchinji. Minor edits to the study plan were instituted following feedback and questions raised in these meetings.

Results

The total 47 health facilities were audited, with 16 in Mchinji and 31 in Zomba (**Table 1**), and 44% (n=531/1197) of clinical staff employed across these facilities were surveyed. All six planned FGDs were completed, with three in each district.

Facility Audits

Availability of seven IMCI drugs was assessed (**Table 2**). Lumefantrine-Artemether and cotrimoxazole were the most available (98% and 96%); while amoxicillin tablets and salbutamol nebuliser solution were the least available (28% and 36%). When available, the majority of drugs were found to be in date. Respiratory rate timers were the least available piece of equipment, with only 8 (17%) facilities across both districts having a functional device. This was followed by pulse oximeters (30%) and micro-nebulisers (32%, **Table 3**). MUAC tapes, scales and mRDTs were almost universally available. Health centres had the highest proportion of non-functional equipment.

Healthcare worker survey

Overall 42% (n=222/531) of the survey respondents reported having IMCI training, with no difference by district (p-value=0.900). Of these, 38% had also received refresher training. The most common group to report training were HSAs (78%), while clinical officers, medical assistants and

Confidential: For Review Only

nurses all reported similar levels (45%), and all attendants except one had no training. Both training and refresher trainings had been most frequently received from NGOs (50% and 64%). The median time since training was 7 years (IQR: 3 – 9).

The mean knowledge score, of a possible 10 points, was 3.96 (range: 0 - 8). Questions on referral decision using clinical scenarios had both the most correct responses (84% - severe dehydration with another sign of severity), and most incorrect (13% - severe dehydration without any other urgent signs). The multivariable regression found a statistically significant association between IMCI knowledge score and having received refresher training (coeff: 0.42; 95% CI: 0.01-0.82) - **Table 4**. However, training alone was not significantly associated with IMCI knowledge. Being a HSA or hospital attendant were both associated with a lower score compared to clinical officers (coeff: -0.82; 95% CI: -1.50, -0.14 and coeff: -1.61; 95% CI: -2.29, -0.92). Amongst those with training, there was a weak but statistically significant positive correlation between knowledge and years since training (correlation coeff: 0.213; p-value: 0.001).

Focus group discussions:

The qualitative data are presented under the following themes: IMCI implementation and practice, barriers to IMCI effectiveness, benefits of IMCI and sustainability.

1. IMCI implementation and practice

This theme includes healthcare workers understanding of IMCI, real-world adaptations, self-perceived quality of implementation, and training received. Overall IMCI was described as a system of assessment, a holistic approach to care for children and as a way to classify illness severity. Across the discussions it was apparent that there were inconsistencies in the implementation of IMCI, but groups generally described a process of weighing, initial clinical assessment, tests and then assigning a diagnosis. One group from Mchinji specifically talked of how a new electronic patient record system had resulted in more structure.

Healthcare workers discussed doing their best to deliver IMCI, in spite of challenges. Although also acknowledged that they do not always follow the guidelines, whether this was intentional was not clear:

> "I guess some people are not using the IMCI approach so it is hard to assess properly. Because they are straight from school sometimes you need updates in order to know when everything is working properly and to give an alarm to the people who are using it" (Zomba, FGD 1)

Confidential: For Review Only

In the groups, there was a mix between those who reported training and those who hadn't received any. Participants in one group described how staff had also received different types of training, depending on who delivered it:

> "There were some people who were trained by the Ministry of health, while others were retained by PSI [an international NGO…] it was a week-long training. But those who were trained by other organisations, the training lasted for three weeks." (Zomba, FGD 2)

2. Barriers to IMCI effectiveness

Three main barriers were discussed: community barriers, lack of adequate resources and lack of trained staff capacity. The issue of caregivers presenting late was a key example raised by several groups as a barrier to effective IMCI implementation. This resulted in tensions around communication and trust between caregivers and providers, and participants were aware of the compounding effects of high patient loads, stock-outs and not having 24-hour staffing.

> "When we ask her the time the child started getting sick she tells a lie by saying they started getting ill today. If we argue her response, you find that sometimes neighbours will confront her by saying the child started suffering sometime back only that she was busy running her business. That's the challenge we encounter sometimes." (Zomba, FDG 3)

While the presence of community structures to support the facilities were described, they noted that they did not always work??? together effectively. In terms of resources, this was raised as a major challenge for some, but not others who described well-functioning supply chain management for essential medications, such as antibiotics. Various scenarios of lacking guidelines, medications, diagnostics and equipment were given to highlight how these limit the ability to provide care for children

> "Sometimes you can find that the child has high fever and there is a need for the child to be given [anti-malarials], but before that you have to confirm with a malaria test, and if you don't have the test kit it becomes a big challenge"(Zomba, FDG 2)

> "The guidelines are not there. Even if you are not trained you can use the guidelines to tell you what to do, but the guidelines are not there" (Zomba, FDG 1)

Multiple dimensions of staffing were raised, from high workloads restricting the amount of time that could be spent conducting an IMCI assessment, not all staff being trained, and lacking supportive

https://mc.manuscriptcentral.com/bmjpo

Confidential: For Review Only

supervision. One discussion with providers from a CHAM hospital however noted that they had no problems with staffing unless there were absences due to events such as funerals.

3. Benefits of IMCI

Benefits of IMCI included better clinical outcomes and better ways of working. Generally, many of the participants acknowledged that IMCI results in improved outcomes for children, and that this also translates into care and care-seeking practices amongst the community.

> "It helps mothers to have additional knowledge of caring [for their] children, through what she is advised at hospital by health workers" (Zomba, FDG 2)

IMCI was described as reducing the workload for hospitals, as frontline facility staff were able to treat conditions such as malaria. The structured and holistic approach was valued by participants in both districts, for example:

> "Yes, it provides a holistic approach to childhood illness, it gives a knowledge on how to treat under-five children. It is a lifesaving system." (Mchinji, FDG 1)

4. IMCI Sustainability

This theme addresses the long term sustainability of IMCI implementation in frontline facilities, with both end-user ownership and sustaining capacity discussed. Notably, this topic was only raised by healthcare providers in Mchinji and included both challenges and concrete recommendations. A key challenge was around the transfer of trained staff, especially when not all staff are sent for IMCI training. Around who attends training, there were concerns around transparency of who is sent for training, which can compound the issue.

> "Yes, it is a problem, for instance someone has gone for IMCI training and next month has been transferred, that means we will have a problem with IMCI because that person didn't finish sharing the information." (Mchinji, FDG 2)

To promote sustainable programming there was a request for healthcare workers to be involved in the development process, and ensuring motivation and follow-up was sustained. NGO instituted programmes were specifically called out for this practice:

https://mc.manuscriptcentral.com/bmjpo

Confidential: For Review Only

"Another problem, it's you NGO people, you don't follow up the programmes" (Mchinji, FDG 3).

Discussion

In this study we explored how IMCI is currently being implemented within two districts in Malawi. We found that there were mixed experiences with both implementation and access to essential supplies, notably with important differences between target infections. In terms of healthcare workers, we found refresher training to be more important in terms of IMCI knowledge than cadre of the healthcare worker and having training alone. These findings pose important topics for further exploration and discussion.

The ability to diagnose and treat respiratory conditions in general, and particularly pneumonia, in frontline facilities was poor. This is especially notable when compared to the almost universal access to malaria RDTs and treatment. ORS, as a treatment for diarrhoeal diseases, was also not readily available at government facilities. The IMCI guidelines recommend seven essential drugs for treating malaria, pneumonia, diarrhoea and ear infections. Several facilities lacked the necessary equipment and first-line treatments to diagnose and treat pneumonia, with the exception of cotrimoxazole, which is a first-line treatment for respiratory infections in children with HIV.

Donor funding for child health has steadily increased since the MDGs were introduced and this has coincided with increased policies for malaria prevention and treatment in Malawi (10). This is reflected in well-funded malaria programmes in Malawi, and in the global funding landscape where malaria has been prioritised through the Global Fund, Bill and Melinda Gates Foundation and USAID (22, 23). This contrasts to pneumonia and diarrhoea, which despite having high burdens, do not receive the same level of strategic funding by large-scale donors. In particular, pneumonia has generally received lower research funding comparative to its global mortality burden (24). This vertical programming focus detracts from the holistic approach to assessing childhood illness, which IMCI promotes.

Resource availability impacts healthcare workers' abilities to adequately perform IMCI assessments and treat according to guidelines. This was apparent from the FGDs with providers, and consistent with previous studies from similar settings, which found better health worker performance in facilities with availability of all essential IMCI equipment (25). Given that some health facilities were better resourced than others, it would be useful to understand different funding allocation, stock management and personnel structures, to ascertain best practices.

https://mc.manuscriptcentral.com/bmjpo

Confidential: For Review Only

The assessment of health care worker IMCI knowledge found more than half of healthcare workers scored under 50%, and those with higher qualifications did not necessarily score better. However, completion of both IMCI training and a refresher course was associated with higher IMCI knowledge. This is in line with other studies on IMCI performance, where healthcare workers who had received IMCI training without a refresher course, performed similarly to those without training (26). It is important to note that while training doesn't necessarily translate into knowledge, knowledge does not always result in adherence to IMCI guidelines and improved practice. A previous study found that those with more experience felt that following the guidelines was not necessary (26). Time constraints and a high work load have also been reported to reduce adherence to IMCI guidelines (27).

We found a weak positive relationship between time since training and knowledge, which may reflect the importance of long-term clinical experience rather than training alone. A systematic review by Rowe et al. (2012) reported that healthcare workers performed better with IMCI training than without, but amongst those trained, over a third of ill children were not treated according to IMCI guidelines (28). Traditional training programs do not necessarily translate to adequate knowledge and skills without ongoing support, and findings from five sub-Saharan African countries found coaching and mentorship improved IMCI service delivery (29). The WHO recommends following up four weeks after the initial IMCI training course to help reinforce the skills learned, and continued supervision (30). There are examples of successful mentorship within child health (31, 32), and community case management in Malawi. However, as lacking supervision and mentorship was raised by healthcare workers in the FGDs, work is needed on effective adoption at frontline facilities.

The qualitative data highlighted a key issue around programme sustainability, and the need for local ownership. Training and refresher courses were often provided by NGOs, not provided to all staff, and the decision for who is trained was not thought to be transparent. Given the frequent transfer of healthcare workers across different facilities, this results in an unequal distribution of workers with IMCI knowledge. It is therefore important that sustainability and handover plans are established by NGOs and research groups, which are agreed up-front with District Health Management Teams.

This study had two key limitations, firstly, that the two districts were selected purposively, and our findings may not be representative to other districts. For example, Mchinji has had multiple research projects in the last decade which have included IMCI refresher trainings and health system strengthening (e.g., McCollum et al. 2016 (33)). In particular, we did not include any district from the northern region. Secondly, the healthcare workers in the survey were those present on the day, and

Confidential: For Review Only

therefore may not cover the full range of providers (e.g., those who predominantly focus on outreach services). The frequency of healthcare worker cadres generally reflected the health workforce composition in Malawi, where HSAs constitute one third of the workforce and few clinical officers (19). The higher proportion of workers from Zomba is also expected given it has a larger population compared to Mchinji, and has traditionally received higher healthcare funding (17).

Malawi's early adoption and scaled-up implementation of IMCI has likely played a key role in the successes made in reducing under-five mortality, but further progress is needed to reach the SDG 3.2 goal. While we found expected challenges in staffing and resources, there are several opportunities for improvement and further research. Firstly, determining which combination of supervision, mentorship and re-training is most cost-effective within the specific context of frontline facilities, to ensure improved knowledge and subsequent quality of care. Secondly, to explore locally adapted IMCI best practices from those facilities which had good supply chain management, positive attitudes to staff transfers and sufficient trained staff capacity. Finally, supporting programming to move away from vertical financing with short term support, to a more holistic approach with embedded sustainability.

Confidential: For Review Only

Acknowledgements

We would like to thank the District Health Office in Mchinji and Zomba for supporting this project, and all the healthcare providers, facility managers, technicians and support start for sharing their time and knowledge. We acknowledge Christopher Kampinga, Tarcizious Phiri, Harry Liyaya (PACHI, Malawi) and Clifford Katumbi (College of Medicine, Malawi) for collecting the audit and survey data.

Funding

This study was funded by a project grant the Swedish Research Council 2017-05579. The funder did not play any role in the design, analysis or interpretation of the data. The analysis and write up was undertaken as part of an MSc student project (the student received a Global Grant Scholarship through Rotary International to undertake a graduate study program).

Conflict of Interest

None declared.

Author Contributions

The study was conceived by HH and CK and study protocols and data collection tools designed together with BZ, CM, LB, ND and AD. Quantitative data collection was overseen by BZ, LB and CM and qualitative data collection was conducted by AD, with support from BZ. Quantitative analysis was conducted by KK with input from CK and HH. Qualitative analysis was conducted by CK and HH, with input from AD and KK. The manuscript was drafted by KK, with significant contributions from CK, HH and AD. All authors read and approved the final manuscript.

Confidential: For Review Only

References

1. The United Nations Children's Fund WHO, World Bank Group, United Nations. Levels and Trends in Child Mortality. Report 2019. New York: UNICEF; 2019.
2. World Health Organisation. Integrated Management of Childhood Illness global survey report. Geneva: World Health Organisation; 2017.
3. GBD 2019 Diseases and Injuries Collaborators. Global burden of 369 diseases and injuries, 1990–2019: a systematic analysis for the Global Burden of Disease Study 2019. The Lancet. 2020.
4. World Health Organisation. Integrated Management of Childhood Illness (IMCI) 2019 [Available from: https://www.who.int/maternal_child_adolescent/topics/child/imci/en/.
5. World Health Organisation. IMIC: Improving Child Health. Geneva: WHO; 1999.
6. Jacobs M, Merson M. Introductory commentary: a strategic review of options for building on lessons learnt from IMCI and iCCM. British Medical Journal. 2018;362.
7. Boschi-Pinto C, Labadie G, Ramachandandran Dilip T, Oliphant N, Dalgish S, Aboubaker S, et al. Global implementation survey of Integrated Management of Childhood Illness (IMCI): 20 years on. BMJ Open. 2018;8.
8. Goga AE, Muhe LM, Forsyth K, Chopra M, Aboubaker S, Martines J, et al. Results of a multi-country exploratory survey of approaches and methods for IMCI case management training. Health Research Policy and Systems. 2009;7(1):18.
9. Nguyen DTK, Leung KK, McIntyre L, Ghali WA, Sauve R. Does Integrated Management of Childhood Illness (IMCI) Training Improve the Skills of Health Workers? A Systematic Review and Meta-Analysis. PLOS ONE. 2013;8(6):e66030.
10. Kanyuka M, Ndawala J, Mleme T, Chisesa L, Makwemba M, Amouzou A, et al. Malawi and Millennium Development Goal 4: a Countdown
to 2015 country case study. The Lancet Global Health. 2016;4:e201-14.
11. Molyneux M, Molyneux E. Reaching Millennium Development 4. The Lancet Global Health. 2016;4(3):PE146-E7.
12. Wemos Foundation. Country Report Malawi. Minding the gap: Who is paying the health workers? Mind the funding gap:. Amsterdam; 2018.
13. Johansson E, Humphreys N, Carvajal-Aquirre L, Amouzou A, Hildenwall H. Determinants of Integrated Management of Childhood Illness (IMCI) non-severe pneumonia classification and care in Malawi health Facilities: Analysis of a national facility census,. Journal of Global Health. 2017;7(2).
14. Bjornstad E, Preidis G, Lufesi N, Olson D, Kamthunzi P, Hosseinipour M, et al. Determining the quality of IMCI pneumonia care in Malawian children. Paediatric International Child Health. 2014;34(1):29-36.
15. National Statistical Office. Malawi Population and Housing Census. Government of Malawi; 2019.
16. National Statistical Office/Malawi, ICF. Malawi Demographic and Health Survey 2015-16. Zomba, Malawi: National Statistical Office and ICF; 2017.
17. Borghi J, Munthali S, Million L, Martinez-Alvarez M. Health financing at district level in Malawi: an analysis of the distribution of funds at two points in time. Health Policy and Planning. 2018;33(1):59-69.
18. Makwero M. Delivery of primary health care in Malawi. African Journal of Primary Health Care & Family Medicine. 2018;10(1).
19. Angwenyi VA, C. Kondowe, K., Zulu Mutchiyeni JK, MCriel, B. Lazarus, J.V., Quinlan T, Bunders-Aelen J. Moving to a strong(er) community health system: analysing the role of community health volunteers in the new national community health strategy
in Malawi. BMJ Global Health. 2018;3:1-10.
20. Quality Assurance Project. Evaluation of an IMCI Computer-based Training Course in Kenya. *Operations Research Results.* . Bethesda, MD: U.S. Agency for International Development (USAID) 2006.

Confidential: For Review Only

21. Gale NK, et al. Using the framework method for the analysis of qualitative data in multi-disciplinary health research. BMC Medical Research Methodology. 2013;13(1):117.
22. USAID. President's Malaria Initiative Malawi Abbreviated Malaria Operational Plan FY 2019.
23. The Global Fund. Results Report. Geneva, Switzerland; 2019.
24. Head M, Fitchett J, Nageshwaran V, Kumari N, Hayward A, Atun R. Research Investments in Global Health: A Systematic Analysis of UK Infectious Disease Research Funding and Global health Metrics, 1997-2013. EBioMedicine. 2016;3:180-90.
25. Pariyo G, Gouws E, Bryce J, Burnham G, Uganda IMCI impact study team. Improving facility-based care for sick children in Uganda: training is not enough. Health Policy and Planning. 2005;20.
26. World Health Organisation. Global Strategy on Human Resources for Health: Workforce 2030 Geneva, Switzerland; 2016.
27. Lange S, Mwisongo A, Mæstad O. Why don't clinicians adhere more consistently to guidelines for the Integrated Management of Childhood Illness (IMCI)? Social Science and Medicine. 2014;104:56-63.
28. Rowe A, Rowe S, Holloway K, Ivanovska V, Mube L, Lambrechts T. Does shortening the training on Integrated Management of Childhood Illness guidelines reduce its effectiveness? A systematic review. Health Policy and Planning. 2012;27:179-93.
29. Manzi A, Hirschhorn L, Sherr K, Chirwa C, Baynes C, Awoonor-Williams J, et al. Mentorship and coaching support strengthening healthcare systems: lessons learned across the five Population Health Implementation and Training partnership projects in sub-Saharan Africa. BMC Health Services Research. 2017;17.
30. World Health Organisation. Guidelines for follow-up after training in the WHO/UNICEF course on Integrated Management of Childhood Illness for first-level health workers. 1999.
31. Asibon A, Lufesi N, Choudhury A, Olvera S, Molyneux E, Oden M, et al. Using a peer mentorship approach improved the use of neonatal continuous positive airway pressure and related outcomes in Malawi. Acta Paediatrica. 2020;109(4):705-10.
32. Guenther T, Mopiwa G, Nsona H, Qazi S, Makuluni R, Fundani CB, et al. Feasibility of implementing the World Health Organization case management guideline for possible serious bacterial infection among young infants in Ntcheu district, Malawi. PLOS ONE. 2020;15(4):e0229248.
33. McCollum ED, King C, Deula R, Zadutsa B, Mankhambo L, Nambiar B, et al. Pulse oximetry for children with pneumonia treated as outpatients in rural Malawi. Bull World Health Organ. 2016;94(12):893-902.

Confidential: For Review Only

Table 1. Health facility and healthcare worker cadre inclusion, by district

**Facilities**	**Total**	**Mchinji**	**Zomba**
Dispensary	7	4	3
Health centre	32	8	24
Rural hospital	3	1	2
CHAM hospital	5	3	2
**Healthcare worker survey respondents**			
Clinical officer	17 (3%)	6 (3%)	11 (3%)
Medical assistant	47 (9%)	15 (8%)	32 (9%)
Nurse/midwife	105 (20%)	32 (17%)	73 (22%)
HSA	190 (36%)	69 (36%)	121 (36%)
Hospital attendant	172 (32%)	71 (37%)	101 (30%)

Confidential: For Review Only

Table 2. *Drug availability and proportion of drugs in date by facility type*

	CHAM hospital (n=5)		Rural hospital (n=3)		Health Centre (n=32)		Dispensary (n=7)	
	**Available (n, %)**	**In date (%)**	**Available (n, %)**	**In date (%)**	**Available (n, %)**	**In date (%)**	**Available (n, %)**	**In date (%)**
Cotrimoxazole tablet	4 (80%)	100%	3 (100%)	100%	32 (100%)	100%	6 (86%)	100%
Amoxicillin tablets	5 (100%)	100%	1 (33%)	100%	6 (19%)	100%	1 (14%)	100%
Amoxicillin syrup	4 (80%)	100%	0	-	23 (71%)	95%	4 (57%)	75%
Lumefantrine-Artemether	5 (100%)	100%	3 (100%)	100%	32 (100%)	100%	6 (86%)	100%
Rectal artesunate	1 (20%)	100%	1 (33%)	100%	19 (59%)	100%	1 (14%)	100%
Oral rehydration salts	5 (100%)	80%	0	-	16 (50%)	100%	3 (43%)	100%
Salbutamol tablets	5 (100%)	100%	2 (67%)	100%	15 (47%)	100%	3 (43%)	100%
Salbutamol nebuliser solution	4 (80%)	100%	1 (33%)	N/A*	5 (16%)	N/A*	7 (100%)	100%
Aminophylline injection**	2 (67%)	100%	1 (100%)	N/A*	3 (38%)	67%	3 (75%)	100%

** Data was missing for these values*

*** Data only collected for Mchinji district (n=16)*

Confidential: For Review Only

Table 3. Equipment Availability by facility type and proportion of functional equipment

	CHAM hospital (n=5)		Rural hospital (n=3)		Health Centre (n=32)		Dispensary (n=7)	
	**Available (n, %)**	**Functional (%)**	**Available (n, %)**	**Functional (%)**	**Available (n, %)**	**Functional (%)**	**Available (n, %)**	**Functional (%)**
Thermometer	5 (100%)	100%	3 (100%)	100%	25 (78%)	96%	7 (100%)	71%
Respiratory rate timer	1 (20%)	100%	0	-	6 (19%)	67%	2	100%
MUAC tape	5 (100%)	100%	3 (100%)	100%	32 (100%)	100%	7 (100%)	100%
Weighing scale	5 (100%)	100%	3 (100%)	100%	32 (100%)	100%	7 (100%)	100%
Micro-nebulizer	3 (60%)	100%	1 (33%)	100%	9 (28%)	78%	2	100%
Malaria RDTs	5 (100%)	100%	3 (100%)	100%	31 (97%)	100%	7 (100%)	100%
Pulse oximeter*	3 (60%)	100%	1 (33%)	100%	10 (31%)	70%	0	-

RDT = rapid diagnostic test; MUAC = mid-upper arm circumference

*Pulse oximetry is recommended but not essential according to IMCI guidelines

Confidential: For Review Only

Table 4. IMCI knowledge score description and multivariate linear regression analysis

VARIABLES	Mean score (n=531)				Coefficient (95% CI)	p-value
	N	Mean	Mchinji (n=193)	Zomba (n=338)		
Training						
No training	309	3.69	3.54	3.77	*Ref*	
IMCI training only	137	4.31	4.40	4.26	0.083 (-0.238, 0.405)	0.611
IMCI training + refresher	85	4.40	4.26	4.50	0.417 (0.012, 0.821)	0.043
Cadre						
Clinical Officer	17	4.76	4.67	4.82	*Ref*	
assistant Medical	47	4.57	4.73	4.50	-0.241 (-0.985, 0.503)	0.526
Nurse/midwife	105	4.62	4.63	4.62	-0.149 (-0.834, 0.536)	0.669
HSA	190	4.13	4.11	4.13	-0.820 (-1.500, -0.142)	0.018
Hospital Attendant	172	3.13	3.04	3.19	-1.605 (-2.286, -0.923)	<0.001
Facility type						
Dispensary	40	3.98	4.29	3.81	*Ref*	
Health Centre	378	4.01	3.91	4.04	0.136 (-0.298, 0.571)	0.538
Rural Hospital	113	3.80	3.76	3.92	-0.004 (-0.501, 0.493)	0.987
District						
Mchinji	193	3.87			*Ref*	
Zomba	338	4.01			0.001 (-0.264, 0.266)	0.993

R-squared: 0.19

Confidential: For Review Only

Appendix 1: Healthcare worker focus group discussion topic guide

1. Current IMCI

- When did you last have IMCI training? Can you describe the training?
- Can you describe what IMCI is?
- Do you think you implement IMCI in your setting?
- Are there any barriers you face in implementing IMCI?
- Are there any benefits you think of implementing IMCI?

2. Current emergency treatment

- Can you give a recent example of child you saw, that you would describe as an emergency?
  - Probe: do others recognise this example, ask for multiple different ones, you can give an example
- Can you describe what you did with this child?
  - Probe: would everyone do the same, what would they do different
- What would you have liked to do in this situation?
- How did you feel about your management of this case?
- How often do you see children like this?
- Do you use any tools or algorithms (guidelines) when assessing emergency cases?
- When managing emergency cases, how do you work? Is it alone, or in a team? Who is part of the team?

3. Current referral procedure

- How often do you refer a child to the hospital? For example, this week how many children has everyone referred?
- Can you describe the process of referral?
  - Probe: documentation, explaining to the caregiver, organising transport
- Do all caregivers complete the referral?
- Are there any barriers you face in referring children?
- Are there any benefits to referring children?

4. ETAT awareness

- How do you decide which order to see patients in your clinic?
- Have you heard of ETAT? Can you describe ETAT?
- Have you ever had training in ETAT? What about other types of triaging?

5. ETAT preparedness

- What steps would need to happen for you implement ETAT?
- Do you have the equipment you need? Can you give examples?
- Do you have the staff you need?
- Are there any barriers to implementing ETAT? And barriers to adequate management of severely ill children?
- Are there any benefits to implementing ETAT in your setting?

Confidential: For Review Only

Appendix 2: Healthcare provider IMCI training and knowledge survey

1.1 Have you ever had IMCI training?	Yes / No	If YES, go to 1.4
1.2 Have you ever been given an orientation or briefing on IMCI?	Yes / No	If NO, go to 1.9
1.3 Who gave you this orientation/ briefing?	1. Facility in-charge<br>2. Other facility staff<br>3. DHMT representative<br>4. Other	Go to 1.9
1.4 When was your first training?	_______ year	
1.5 Who provided this first training?	1. MoH/DHMT<br>2. NGO<br>3. Research project<br>4. During school/training<br>5. Other	
1.6 Have you had a refresher training or active mentorship since?	Y / N	If NO, go to 1.9
1.7 When was the refresher?	_______ year	
1.8 Who provided this refresher training?	1. MoH / DHTM<br>2. NGO<br>3. Research project<br>4. During school/training<br>5. Other	Select all that apply
1.9 What is a child's classification if he is 10 months old, has had a cough that lasted two days, has a breathing rate of 46 breaths per minute and chest indrawing?[1]	1. Cough or cold<br>2. Pneumonia<br>3. Severe pneumonia<br>4. Very severe febrile disease<br>5. Don't know	Select one
1.10 What are the four main symptoms for which every sick child should be checked?	1. Malnutrition, cough, vitamin A, ear problems<br>2. Anemia, fever, diarrhea, ear problem<br>3. Cough, diarrhea, malnutrition, ear problem<br>4. Cough, diarrhea, fever, ear problem<br>5. Don't know	Select the best answer
1.11 For each of the following cases, select **Yes** if urgent referral is needed or select **No** if urgent referral is not needed.		
1.11a A 6-month-old boy does not have general danger signs. He is classified with: MASTOIDITIS and NO ANEMIA AND NOT VERY LOW WEIGHT	1. Yes<br>2. No<br>3. Don't know	
1.11b A 7-month-old girl does not have general danger signs. She is classified with: COUGH and DIARRHEA	1. Yes<br>2. No<br>3. Don't know	

Confidential: For Review Only

1.11c A 9-month-old boy is lethargic. He is classified with: DIARRHEA and WITH SEVERE DEHYDRATION	1. Yes<br>2. No<br>3. Don't know	
1.11d A 2-year-old girl does not have general danger signs. She is classified with: DIARRHEA and SEVERE DEHYDRATION and SEVERE MALNUTRITION	1. Yes<br>2. No<br>3. Don't know	
1.12 If a child has any of the four general danger signs, you should urgently refer him to hospital for treatment, These signs are:	1. Unable to drink/feed<br>2. Severe cough<br>3. Convulsions<br>4. Vomiting everything<br>5. Lethargy / unconsciousness<br>6. Bloody stools<br>7. Don't know	Select four from the list
1.13 According to IMCI, a mother of a sick child should be counselled about what topics:	1. Importance of fluids and feeding<br>2. When to return to the clinic immediately<br>3. Her own health<br>4. Immunisation<br>5. When to return for a follow-up visit<br>6. The treatments being given to the child<br>7. Family planning<br>8. Don't know	Select all that apply
1.14 What are two signs that are used to classify severe malnutrition	1. Small arm circumference<br>2. Visible severe wasting<br>3. Oedema of both feet<br>4. Severe dehydration<br>5. Don't know	Select two from the list
1.15 To classify the dehydration status of a child with diarrhoea you will look:	1. At the general condition<br>2. For sunken eyes<br>3. For oedema of both feet<br>4. If the child is drinking eagerly<br>5. For palmar pallor<br>6. For a swollen abdomen<br>7. Don't know	Select all that apply

Confidential: For Review Only

BMJ Paediatrics Open

Integrated Management of Childhood Illnesses (IMCI): A mixed-methods study on implementation, knowledge and resource availability in Malawi

Journal:	*BMJ Paediatrics Open*
Manuscript ID	bmjpo-2021-001044.R1
Article Type:	Original research
Date Submitted by the Author:	05-Apr-2021
Complete List of Authors:	Kilov, Kim; Karolinska Institute, Department of Global Public Health<br>Hildenwall, Helena; Karolinska Institute, Department of Global Public Health<br>Dube, Albert; Malawi Epidemiology and Intervention Research Unit (MEIRU)<br>Zadutsa, Beatiwel; Parent and Child Health Initiative<br>Banda, Lumbani; Parent and Child Health Initiative<br>Langton, Josephine; University of Malawi College of Medicine, Paediatrics<br>Desmond, Nicola; Malawi-Liverpool-Wellcome Trust Programme<br>Lufesi, Norman; Ministry of Health<br>Makwenda, Charles; Parent and Child Health Initiative<br>King, Carina; University College London, Institute for Global Health; Karolinska Institute, Department of Global Public Health
Keywords:	Health services research

SCHOLARONE™
Manuscripts

I, the Submitting Author has the right to grant and does grant on behalf of all authors of the Work (as defined in the below author licence), an exclusive licence and/or a non-exclusive licence for contributions from authors who are: i) UK Crown employees; ii) where BMJ has agreed a CC-BY licence shall apply, and/or iii) in accordance with the terms applicable for US Federal Government officers or employees acting as part of their official duties; on a worldwide, perpetual, irrevocable, royalty-free basis to BMJ Publishing Group Ltd ("BMJ") its licensees and where the relevant Journal is co-owned by BMJ to the co-owners of the Journal, to publish the Work in this journal and any other BMJ products and to exploit all rights, as set out in our licence.

The Submitting Author accepts and understands that any supply made under these terms is made by BMJ to the Submitting Author unless you are acting as an employee on behalf of your employer or a postgraduate student of an affiliated institution which is paying any applicable article publishing charge ("APC") for Open Access articles. Where the Submitting Author wishes to make the Work available on an Open Access basis (and intends to pay the relevant APC), the terms of reuse of such Open Access shall be governed by a Creative Commons licence – details of these licences and which Creative Commons licence will apply to this Work are set out in our licence referred to above.

Other than as permitted in any relevant BMJ Author's Self Archiving Policies, I confirm this Work has not been accepted for publication elsewhere, is not being considered for publication elsewhere and does not duplicate material already published. I confirm all authors consent to publication of this Work and authorise the granting of this licence.

Confidential: For Review Only

Title: Integrated Management of Childhood Illnesses (IMCI): A mixed-methods study on implementation, knowledge and resource availability in Malawi

Authors: Kim Kilov[1], Helena Hildenwall[1,2,3], Albert Dube[4], Beatiwel Zadutsa[5], Lumbani Banda[5], Josephine Langton[6], Nicola Desmond[7], Norman Lufesi[8], Charles Makwenda[5], Carina King[1,9]*

Affiliations:

1. Department of Global Public Health, Karolinksa Institutet, Stockholm, Sweden
2. Astrid Lindgren Children's Hospital, Karolinska University Hospital, Stockholm, Sweden
3. Department of Clinical Science, Intervention and Technology, Karolinska Institutet, Stockholm, Sweden
4. Malawi Epidemiology and Intervention Research Unit (MEIRU), Lilongwe, Malawi
5. Parent and Child Health Initiative, Lilongwe, Malawi
6. Department of Paediatrics, College of Medicine, Blantyre, Malawi
7. Social Science Unit, Malawi-Liverpool-Wellcome Trust Programme, Blantyre, Malawi
8. Acute Respiratory Infections Unit, Ministry of Health, Lilongwe, Malawi
9. Institute for Global Health, University College London, London, UK

***Corresponding author:**

Department of Global Public Health, Karolinska Institutet, Widerströmska huset, Tomtebodavägen 18B, 171 65 Solna, Sweden. Email: carina.king@ki.se

Keywords:

Integrated Management of Childhood Illness; Malawi; Under-five mortality; Health worker; Knowledge; Resources

Word count: 3644

Confidential: For Review Only

Abstract

Background

The introduction of the World Health Organisation's Integrated Management of Childhood Illnesses guidelines (IMCI) in the mid-1990s contributed to global reductions in under-five mortality. However, issues in quality of care have been reported. We aimed to determine resource availability and healthcare worker knowledge of IMCI guidelines in two Malawian districts.

Methods

We conducted a mixed-method study, including health facility audits, healthcare provider survey and focus group discussions (FGDs) with facility staff. The study was conducted between January to April 2019 in Mchinji (central region) and Zomba (southern region) districts. Quantitative data was described using proportions and chi-squared tests; linear regression was conducted to explore factors associated with IMCI knowledge. Qualitative data was analysed using a pragmatic framework approach. Qualitative and quantitative data were analysed and presented separately.

Results

Forty-seven health facilities and 531 healthcare workers were included. Lumefantrine-Artemether and cotrimoxazole were the most available drugs (98% and 96%); while amoxicillin tablets and salbutamol nebuliser solution were the least available (28% and 36%). Respiratory rate timers were the least available piece of equipment, with only 8 (17%) facilities having a functional device. The mean IMCI knowledge score was 3.96 out of 10, and there was a statistically significant association between knowledge and having received refresher training (coeff: 0.42; 95% CI: 0.01-0.82). Four themes were identified in the FGDs: IMCI implementation and practice, barriers to IMCI, benefits of IMCI and sustainability.

Conclusion

We found key gaps in IMCI implementation, however these were not homogenous across facilities, suggesting opportunities to learn from locally adapted IMCI best practices. Improving on-going mentorship, training and supervision should be explored to improve quality of care, and programming which moves away from vertical financing with short term support, to a more holistic approach with embedded sustainability may address the balance of resources for different conditions.

Confidential: For Review Only

Summary Box:

What is known about the subject

- WHO's integrated management of childhood illness guidelines have been important in reducing child deaths in low-resource settings
- However, quality of care issues in IMCI implementation have been noted, including lack of resources, drugs and trained staff
- Training in IMCI is variable, and recommendations for and evidence on the impact of refresher trainings is limited

What this study adds

- In two Malawian district, we found key IMCI implementation gaps; these were not homogenous across facilities suggesting opportunities to learn from locally adapted best practice
- Training alone was not independently associated with improved IMCI knowledge, but having had refresher training was.
- Resource gaps were more prominent for respiratory and diarrhoeal case management than malaria, highlighting issues in vertical financing

Confidential: For Review Only

Introduction

Despite a reduction in under five deaths globally from 12.5 million in 1990 to 5.3 million in 2018, progress has been uneven (1). The highest burden is seen in Sub-Saharan Africa, with considerable national and sub-national variation (1). A key strategy in the success to date was the introduction of the WHOs Integrated Management of Childhood Illness (IMCI) guidelines (2). At the time, the major causes of child mortality were pneumonia, malaria, measles, malnutrition and diarrhoea (2). More recently this has shifted to neonatal complications (1), while pneumonia remains the leading infectious cause of mortality in children under-five (3).

IMCI was intended for countries with under-five mortality rates higher than 40 per 1000 live births, and focuses on three components: improving case management skills, strengthening health systems and improving community health practices (4, 5). While IMCI has been partially or fully adopted by 100 countries (6, 7), a survey from 2016 reported only 44 countries were considered to be fully implementing it (2). Further, IMCI coverage was lowest in countries with the highest mortality rates; of the 26 countries to achieve the Millennium Development Goal 4 - reducing under-five mortality by two thirds, 20 had fully implemented IMCI (2).

Key strengths of IMCI are the holistic approach, rational use of medications and improved quality and efficiency of health service provision (2, 7). However, implementation barriers and inconsistencies have been reported. For example, the WHO guidance for training contains 7 modules taught over 11 days, but local adaptations have led to considerable differences, with examples of both shorter and distance learning approaches (8, 9). Currently there is a lack of standardised guidance on the frequency, content, pedagogical approach and expected learning outcomes for refresher courses.

Malawi was an early adopter of IMCI, first implementing it in 2000 (10), and was one of only 10 low-income countries in Sub-Saharan Africa to achieve MDG4 (11). This success has been attributed to proactive policies, scale-up and introduction of vaccinations, coverage of insecticide treated bed nets, IMCI and integrated community case management (10). However, Malawi is not currently on track to achieve the target set out in Sustainable Development Goal 3.2, and faces considerable challenges with an under-resourced health sector with workforce shortages (12). Previous studies have reported suboptimal IMCI implementation for pneumonia, with issues in clinical assessments, quality of diagnosis and antibiotic prescription (13, 14).

Given changes in causes of under-five mortality, varied successes in IMCI implementation and ongoing challenges of under-resourced health systems, it is important to assess if IMCI is effectively supported and implemented. We aimed to describe current IMCI implementation at primary care in

Confidential: For Review Only

Malawi, and determine whether there were sufficient resources and trained staff available to assess and manage children under-five according to these guidelines.

Methods

We conducted a concurrent mixed-methods study, covering all dispensaries, health centres, rural hospitals and Christian Health Association of Malawi (CHAM) hospitals in Zomba and Mchinji districts. Data was collected between January – April 2019. The study included facility audits to assess resource availability, healthcare provider surveys to assess training and knowledge, and focus groups discussions (FGDs) with healthcare providers and facility managers to explore current implementation. Qualitative and quantitative data were analysed and are presented separately.

Setting

Mchinji district is in Malawi's central region, with an under-five population of approximately 90,000 and an under-five mortality rate of 123/1,000 livebirths in the 2015-16 DHS. Zomba is located in the Southern region, with an under-five population of 120,000 and mortality of 54/1,000 livebirths (15, 16). Zomba has a larger urban population and has historically received both higher per capita domestic and external funding (15, 17), while Mchinji has 84% of the population living rurally (15). The health system is made up of three levels (18). Health centres, dispensaries and rural clinics deliver primary care and are linked to community care via Health Surveillance Assistants (HSA) (19). Secondary care is delivered at district hospitals and regional referral hospitals provide tertiary care. A summary of healthcare workers and their roles are presented in Web-Appendix 1. Government care is free, and costs of services delivered by CHAM facilities are subsidised through service level agreements resulting in small out of pocket payments (21).

Sampling

All dispensaries, health centres, rural and CHAM hospitals (i.e., frontline facilities) were eligible to be audited. A convenience sample of staff were recruited for the survey, including staff who interact with paediatric patients and were present at the facility at the time of data collection. Qualitative participants were selected using purposive sampling, aiming to conduct three FGDs in each district. Participants were invited to participate by phone by a member of study staff, with permission from the District Health Management Team and senior HSAs. The groups targeted included: 1. HSAs and attendants at health centres; 2. medical assistants and nurses at health centres; 3. clinical officers and medical assistants at rural hospitals. We invited up to 10 participants per group.

Confidential: For Review Only

Quantitative Data Collection

The audit assessed the number of healthcare workers, availability and functionality of essential IMCI equipment (thermometer, respiratory rate timer, mid-upper arm circumference (MUAC) tape, scale, nebuliser) and availability of IMCI drugs (antibiotics, anti-malarials, oral rehydration solution and salbutamol) used for outpatient case management and pre-referral treatment, and whether they were in date. Data on drugs and equipment used for complex emergency cases are not presented. The structured healthcare worker survey included questions on: cadre, previous IMCI training, refresher courses, years since training and refresher training received and knowledge of the 2014 IMCI guidelines. The knowledge questions were adapted from an IMCI computer-based training course evaluation delivered by USAID in Kenya (20); this was selected due to its short length covering different elements of IMCI knowledge, and the questions should not have been previously seen by participants in our setting.

Data was collected by trained study staff who visited each facility at a pre-arranged time to conduct the audit and surveys. The study staff consisted of clinical officers and monitoring and evaluation officers. The data was collected with support from facility in-charge, pharmacy and health management information staff and included visual inspection of drug stocks to check quantities and expiry dates, equipment functionality and closed questions. Visual inspection by the study staff member was conducted to reduce potential recall and social desirability biases. Data was entered into android tablets using Open Data Kit Collect, with in-built cleaning and skip-pattern rules to promote data quality. The survey was self-completed on an android tablet for healthcare workers who were familiar with smart phone technology. If unfamiliar, the survey was interviewer administered.

Qualitative Data Collection

FGDs were held at healthcare facilities and led by a male Malawian researcher (AD) with experience of qualitative research, supported by a clinical officer to provide IMCI specific knowledge. Discussions were open to be conducted in Chichewa or English, depending on the content and preference of participants. Interviews and discussions were audio-recorded, transcribed and where necessary translated into English. Participants were reimbursed for their travel expenses and provided with refreshments.

Analysis

The audit data was described using proportions, and compared between facility types and districts using Chi-squared tests. Healthcare worker knowledge was summarised into a score with a

https://mc.manuscriptcentral.com/bmjpo

Confidential: For Review Only

maximum of 10 points. Mean scores were compared for the following independent variables: IMCI training received, years since training, refresher training received, years since refresher training, cadre, facility type and district. Multivariable linear regression analysis was conducted to assess the association between IMCI knowledge scores and training. The following confounders were considered: district (given different funding levels), facility type, health worker qualification and refresher course. Quantitative analysis was performed using Stata IC 16.0.

FGDs were analysed using a pragmatic framework approach, with pre-defined themes based on the topic guide (21) (Appendix 2). Emergent themes were coded during the analysis. Discrepancies were agreed through discussion, and the interpretation and conclusions shared and discussed with the wider study team. Coding was done by HH, with a sub-set double coded by CK. These codes were then discussed, refined and organised into themes, which were checked by AD for consistency with his interpretation and the local context.

__Patient and Public Involvement__

Patients and public were not involved in the design or execution of the study. Prior to starting, the protocol was presented to both District Health Management and District Executive Committees (which include local government and civil society representation) in both Zomba and Mchinji. Minor edits to the study plan were instituted following feedback and questions raised in these meetings.

Results

The total 47 health facilities were audited, with 16 in Mchinji and 31 in Zomba (**Table 1**), and 44% (n=531/1197) of clinical staff employed across these facilities were surveyed. All six planned FGDs were completed, with three in each district.

__Facility Audits__

Availability of seven IMCI drugs was assessed (**Table 2**). Lumefantrine-Artemether and cotrimoxazole were the most available (98% and 96%); while amoxicillin tablets and salbutamol nebuliser solution were the least available (28% and 36%). When available, the majority of drugs were found to be in date. Respiratory rate timers were the least available piece of equipment, with only 8 (17%) facilities across both districts having a functional device. This was followed by pulse oximeters (30%) and micro-nebulisers (32%, **Table 3**). MUAC tapes, scales and mRDTs were almost universally available. Health centres had the highest proportion of non-functional equipment.

__Healthcare worker survey__

Confidential: For Review Only

Overall 42% (n=222/531) of the survey respondents reported having IMCI training, with no difference by district (p-value=0.900). Of these, 38% had also received refresher training. The most common group to report training were HSAs (78%), while clinical officers, medical assistants and nurses all reported similar levels (45%), and only one attendant reported training. Training was most frequently received from NGOs (50%) followed by Ministry of Health (36%) and during qualification training (14%); refresher trainings were delivered by NGOs (64%) and Ministry of Health (36%). The median time since initial training was 7 years (95% confidence interval: 6, 8; range: 0 - 21).

The mean knowledge score, of a possible 10 points, was 3.96 (95% confidence interval: 3.83, 4.08; range: 0 – 8), with 65% scoring less than 5 (Web Appendix 3). Questions on referral decision using clinical scenarios had both the most correct responses (84% - severe dehydration with another sign of severity), and most incorrect (13% - severe dehydration without any other urgent signs). The multivariable regression found a statistically significant association between IMCI knowledge score and having received refresher training (coeff: 0.42; 95% CI: 0.01-0.82) - **Table 4**. However, training alone was not significantly associated with IMCI knowledge. Being a HSA or hospital attendant were both associated with a lower score compared to clinical officers (coeff: -0.82; 95% CI: -1.50, -0.14 and coeff: -1.61; 95% CI: -2.29, -0.92). Amongst those with training, there was a weak but statistically significant positive correlation between knowledge and years since training (correlation coeff: 0.213; p-value: 0.001).

Focus group discussions:

The qualitative data are presented under the following themes: IMCI implementation and practice, barriers to IMCI effectiveness, benefits of IMCI and sustainability.

1. IMCI implementation and practice

This theme includes healthcare workers understanding of IMCI, real-world adaptations, self-perceived quality of implementation, and training received. Overall IMCI was described as a system of assessment, a holistic approach to care for children and as a way to classify illness severity. Across the discussions it was apparent that there were inconsistencies in the implementation of IMCI, but groups generally described a process of weighing, initial clinical assessment, tests and then assigning a diagnosis. One group from Mchinji specifically talked of how a new electronic patient record system had resulted in more structure.

Healthcare workers discussed doing their best to deliver IMCI, in spite of challenges. Although also acknowledged that they do not always follow the guidelines, whether this was intentional was not clear:

Confidential: For Review Only

"I guess some people are not using the IMCI approach so it is hard to assess properly. Because they are straight from school sometimes you need updates in order to know when everything is working properly and to give an alarm to the people who are using it" (Zomba, FGD 1)

In the groups, there was a mix between those who reported training and those who hadn't received any. Participants in one group described how staff had also received different types of training, depending on who delivered it:

"There were some people who were trained by the Ministry of health, while others were retained by PSI [an international NGO…] it was a week-long training. But those who were trained by other organisations, the training lasted for three weeks." (Zomba, FGD 2)

2. Barriers to IMCI effectiveness

Three main barriers were discussed: community barriers, lack of adequate resources and lack of trained staff capacity. The issue of caregivers presenting late was a key example raised by several groups as a barrier to effective IMCI implementation. This resulted in tensions around communication and trust between caregivers and providers, and participants were aware of the compounding effects of high patient loads, stock-outs and not having 24-hour staffing.

"When we ask her the time the child started getting sick she tells a lie by saying they started getting ill today. If we argue her response, you find that sometimes neighbours will confront her by saying the child started suffering sometime back only that she was busy running her business. That's the challenge we encounter sometimes." (Zomba, FDG 3)

While the presence of community structures to support the facilities were described, they noted that they did not always work together effectively. In terms of resources, this was raised as a major challenge for some, but not others who described well-functioning supply chain management for essential medications, such as antibiotics. Various scenarios of lacking guidelines, medications, diagnostics and equipment were given to highlight how these limit the ability to provide care for children

"Sometimes you can find that the child has high fever and there is a need for the child to be given [anti-malarials], but before that you have to confirm with a malaria test, and if you don't have the test kit it becomes a big challenge"(Zomba, FDG 2)

Confidential: For Review Only

> "The guidelines are not there. Even if you are not trained you can use the guidelines to tell you what to do, but the guidelines are not there" (Zomba, FDG 1)

Multiple dimensions of staffing were raised, from high workloads restricting the amount of time that could be spent conducting an IMCI assessment, not all staff being trained, and lacking supportive supervision. One discussion with providers from a CHAM hospital however noted that they had no problems with staffing unless there were absences due to events such as funerals.

3. Benefits of IMCI

Benefits of IMCI included better clinical outcomes and better ways of working. Generally, many of the participants acknowledged that IMCI results in improved outcomes for children, and that this also translates into care and care-seeking practices amongst the community.

> "It helps mothers to have additional knowledge of caring [for their] children, through what she is advised at hospital by health workers" (Zomba, FDG 2)

IMCI was described as reducing the workload for hospitals, as frontline facility staff were able to treat conditions such as malaria. The structured and holistic approach was valued by participants in both districts, for example:

> "Yes, it provides a holistic approach to childhood illness, it gives a knowledge on how to treat under-five children. It is a lifesaving system." (Mchinji, FDG 1)

4. IMCI Sustainability

This theme addresses the long term sustainability of IMCI implementation in frontline facilities, with both end-user ownership and sustaining capacity discussed. Notably, this topic was only raised by healthcare providers in Mchinji and included both challenges and concrete recommendations. A key challenge was around the transfer of trained staff, especially when not all staff are sent for IMCI training. Around who attends training, there were concerns around transparency of who is sent for training, which can compound the issue.

> "Yes, it is a problem, for instance someone has gone for IMCI training and next month has been transferred, that means we will have a problem with IMCI because that person didn't finish sharing the information." (Mchinji, FDG 2)

Confidential: For Review Only

To promote sustainable programming there was a request for healthcare workers to be involved in the development process, and ensuring motivation and follow-up was sustained. NGO instituted programmes were specifically called out for this practice:

> "Another problem, it's you NGO people, you don't follow up the programmes" (Mchinji, FDG 3).

Discussion

In this study we explored how IMCI is currently being implemented within two districts in Malawi. We found that there were mixed experiences with both implementation and access to essential supplies, notably with important differences between target infections. In terms of healthcare workers, we found refresher training to be more important in terms of IMCI knowledge than cadre of the healthcare worker and having training alone. These findings pose important topics for further exploration and discussion.

The ability to diagnose and treat respiratory conditions in general, and particularly pneumonia, in frontline facilities was poor. This is especially notable when compared to the almost universal access to malaria RDTs and treatment. ORS, as a treatment for diarrhoeal diseases, was also not readily available at government facilities. The IMCI guidelines recommend seven essential drugs for treating malaria, pneumonia, diarrhoea and ear infections. Several facilities lacked the necessary equipment and first-line treatments to diagnose and treat pneumonia, with the exception of cotrimoxazole, which is a first-line treatment for respiratory infections in children with HIV.

Donor funding for child health has steadily increased since the MDGs were introduced in 2000 and this has coincided with increased policies for malaria prevention and treatment in Malawi (10). This is reflected in well-funded malaria programmes in Malawi (Malawi Malaria Communication Strategy 2015-2020 (22)), and in the global funding landscape where malaria has been prioritised through the Global Fund, Bill and Melinda Gates Foundation and USAID (23, 24). This contrasts to pneumonia and diarrhoea, which despite having high burdens, do not receive the same level of strategic funding by large-scale donors. In particular, pneumonia has generally received lower research funding comparative to its global mortality burden (25). This vertical programming focus is often disease specific and detracts from the holistic approach to assessing childhood illness, which IMCI promotes. Further exploration of factors which promote sustainability at different levels of the health system could reveal important insights for future programming.

https://mc.manuscriptcentral.com/bmjpo

Confidential: For Review Only

Resource availability impacts healthcare workers' abilities to adequately perform IMCI assessments and treat according to guidelines. This was apparent from the FGDs with providers, and consistent with previous studies from similar settings, which found better health worker performance in facilities with availability of all essential IMCI equipment (26). Given that some health facilities were better resourced than others, it would be useful to understand different funding allocation, stock management and personnel structures, to ascertain best practices.

The assessment of health care worker IMCI knowledge found more than half of healthcare workers scored under 50%, and those with higher qualifications did not necessarily score better. Completion of both IMCI training and a refresher course was associated with higher IMCI knowledge. This is in line with other studies on IMCI performance, where healthcare workers who had received IMCI training without a refresher course, performed similarly to those without training (27). It is important to note that while training doesn't necessarily translate into knowledge, knowledge does not always result in adherence to IMCI guidelines and improved practice. A previous study found that those with more experience felt that following the guidelines was not necessary (27). Time constraints and a high work load have also been reported to reduce adherence to IMCI guidelines (28).

We found a weak positive relationship between time since training and knowledge, which may reflect the importance of long-term clinical experience rather than training alone. A systematic review by Rowe et al. (2012) reported that healthcare workers performed better with IMCI training than without, but amongst those trained, over a third of ill children were not treated according to IMCI guidelines (29). Traditional training programs do not necessarily translate to adequate knowledge and skills without ongoing support, and findings from five sub-Saharan African countries found coaching and mentorship improved IMCI service delivery (30). The WHO recommends following up four weeks after the initial IMCI training course to help reinforce the skills learned, and continued supervision (31). There are examples of successful mentorship within child health (32, 33), and community case management in Malawi. However, as lacking supervision and mentorship was raised by healthcare workers in the FGDs, work is needed on effective adoption at frontline facilities.

The qualitative data highlighted a key issue around programme sustainability, and the need for local ownership. Training and refresher courses were often provided by NGOs (through external funding), not provided to all staff, and the decision for who is trained was not thought to be transparent. Given the frequent transfer of healthcare workers across different facilities, this results in an unequal distribution of workers with IMCI knowledge. It is therefore important that sustainability

Confidential: For Review Only

and handover plans are established by NGOs and research groups, which are agreed up-front with District Health Management Teams.

This study had three key limitations, firstly, that the two districts were selected purposively, and our findings may not be representative to other districts. For example, Mchinji has had multiple research projects in the last decade which have included IMCI refresher trainings and health system strengthening (e.g., McCollum et al. 2016 (34)). In particular, we did not include any district from the northern region. Secondly, the healthcare workers in the survey were those present on the day, and therefore may not cover the full range of providers (e.g., those who predominantly focus on outreach services). The frequency of healthcare worker cadres generally reflected the health workforce composition in Malawi, where HSAs constitute one third of the workforce and few clinical officers (19). The higher proportion of workers from Zomba is also expected given it has a larger population compared to Mchinji, and has traditionally received higher healthcare funding (17). Finally, assessment of practice was not performed, with direct observations of health worker performance posing several ethical and methodological challenges (e.g. the Hawthorne effect (35)). We therefore cannot comment on quality of IMCI care provision.

Malawi's early adoption and scaled-up implementation of IMCI has likely played a key role in the successes made in reducing under-five mortality, but further progress is needed to reach the SDG 3.2 goal. While we found expected challenges in staffing and resources, there are several opportunities for improvement and further research. Firstly, determining which combination of supervision, mentorship and re-training is most cost-effective within the specific context of frontline facilities, to ensure improved knowledge and subsequent quality of care. Secondly, to explore locally adapted IMCI best practices from those facilities which had good supply chain management, positive attitudes to staff transfers and sufficient trained staff capacity. Finally, supporting programming to move away from vertical financing with short term support, to the more holistic approach which IMCI promotes and considers strategies for embedded sustainability.

Confidential: For Review Only

Acknowledgements

We would like to thank the District Health Office in Mchinji and Zomba for supporting this project, and all the healthcare providers, facility managers, technicians and support start for sharing their time and knowledge. We acknowledge Christopher Kampinga, Tarcizious Phiri, Harry Liyaya (PACHI, Malawi) and Clifford Katumbi (College of Medicine, Malawi) for collecting the audit and survey data.

Funding

This study was funded by a project grant the Swedish Research Council 2017-05579. The funder did not play any role in the design, analysis or interpretation of the data. The analysis and write up was undertaken as part of an MSc student project (the student received a Global Grant Scholarship through Rotary International to undertake a graduate study program).

Conflict of Interest

None declared.

Author Contributions

The study was conceived by HH and CK and study protocols and data collection tools designed together with BZ, CM, LB, ND and AD. Quantitative data collection was overseen by BZ, LB and CM and qualitative data collection was conducted by AD, with support from BZ. Quantitative analysis was conducted by KK with input from CK and HH. Qualitative analysis was conducted by CK and HH, with input from AD and KK. The manuscript was drafted by KK, with significant contributions from CK, HH and AD. All authors read and approved the final manuscript.

Confidential: For Review Only

References

1. The United Nations Children's Fund WHO, World Bank Group, United Nations. Levels and Trends in Child Mortality. Report 2019. New York: UNICEF; 2019.
2. World Health Organisation. Integrated Management of Childhood Illness global survey report. Geneva: World Health Organisation; 2017.
3. GBD 2019 Diseases and Injuries Collaborators. Global burden of 369 diseases and injuries, 1990–2019: a systematic analysis for the Global Burden of Disease Study 2019. The Lancet. 2020.
4. World Health Organisation. Integrated Management of Childhood Illness (IMCI) 2019 [Available from: https://www.who.int/maternal_child_adolescent/topics/child/imci/en/.
5. World Health Organisation. IMIC: Improving Child Health. Geneva: WHO; 1999.
6. Jacobs M, Merson M. Introductory commentary: a strategic review of options for building on lessons learnt from IMCI and iCCM. British Medical Journal. 2018;362.
7. Boschi-Pinto C, Labadie G, Ramachandandran Dilip T, Oliphant N, Dalgish S, Aboubaker S, et al. Global implementation survey of Integrated Management of Childhood Illness (IMCI): 20 years on. BMJ Open. 2018;8.
8. Goga AE, Muhe LM, Forsyth K, Chopra M, Aboubaker S, Martines J, et al. Results of a multi-country exploratory survey of approaches and methods for IMCI case management training. Health Research Policy and Systems. 2009;7(1):18.
9. Nguyen DTK, Leung KK, McIntyre L, Ghali WA, Sauve R. Does Integrated Management of Childhood Illness (IMCI) Training Improve the Skills of Health Workers? A Systematic Review and Meta-Analysis. PLOS ONE. 2013;8(6):e66030.
10. Kanyuka M, Ndawala J, Mleme T, Chisesa L, Makwemba M, Amouzou A, et al. Malawi and Millennium Development Goal 4: a Countdown to 2015 country case study. The Lancet Global Health. 2016;4:e201-14.
11. Molyneux M, Molyneux E. Reaching Millennium Development 4. The Lancet Global Health. 2016;4(3):PE146-E7.
12. Wemos Foundation. Country Report Malawi. Minding the gap: Who is paying the health workers? Mind the funding gap:. Amsterdam; 2018.
13. Johansson E, Humphreys N, Carvajal-Aquirre L, Amouzou A, Hildenwall H. Determinants of Integrated Management of Childhood Illness (IMCI) non-severe pneumonia classification and care in Malawi health Facilities: Analysis of a national facility census,. Journal of Global Health. 2017;7(2).
14. Bjornstad E, Preidis G, Lufesi N, Olson D, Kamthunzi P, Hosseinipour M, et al. Determining the quality of IMCI pneumonia care in Malawian children. Paediatric International Child Health. 2014;34(1):29-36.
15. National Statistical Office. Malawi Population and Housing Census. Government of Malawi; 2019.
16. National Statistical Office/Malawi, ICF. Malawi Demographic and Health Survey 2015-16. Zomba, Malawi: National Statistical Office and ICF; 2017.
17. Borghi J, Munthali S, Million L, Martinez-Alvarez M. Health financing at district level in Malawi: an analysis of the distribution of funds at two points in time. Health Policy and Planning. 2018;33(1):59-69.
18. Makwero M. Delivery of primary health care in Malawi. African Journal of Primary Health Care & Family Medicine. 2018;10(1).
19. Angwenyi VA, C. Kondowe, K., Zulu Mutchiyeni JK, MCriel, B. Lazarus, J.V., Quinlan T, Bunders-Aelen J. Moving to a strong(er) community health system: analysing the role of community health volunteers in the new national community health strategy in Malawi. BMJ Global Health. 2018;3:1-10.
20. Quality Assurance Project. Evaluation of an IMCI Computer-based Training Course in Kenya. *Operations Research Results.* . Bethesda, MD: U.S. Agency for International Development (USAID) 2006.

Confidential: For Review Only

21. Gale NK, et al. Using the framework method for the analysis of qualitative data in multi-disciplinary health research. BMC Medical Research Methodology. 2013;13(1):117.
22. Ministry of Health Malawi. Malawi Malaria Communication Strategy 2015-2020. 2015.
23. USAID. President's Malaria Initiative Malawi Abbreviated Malaria Operational Plan FY 2019.
24. The Global Fund. Results Report. Geneva, Switzerland; 2019.
25. Head M, Fitchett J, Nageshwaran V, Kumari N, Hayward A, Atun R. Research Investments in Global Health: A Systematic Analysis of UK Infectious Disease Research Funding and Global health Metrics, 1997-2013. EBioMedicine. 2016;3:180-90.
26. Pariyo G, Gouws E, Bryce J, Burnham G, Uganda IMCI impact study team. Improving facility-based care for sick children in Uganda: training is not enough. Health Policy and Planning. 2005;20.
27. World Health Organisation. Global Strategy on Human Resources for Health: Workforce 2030 Geneva, Switzerland; 2016.
28. Lange S, Mwisongo A, Mæstad O. Why don't clinicians adhere more consistently to guidelines for the Integrated Management of Childhood Illness (IMCI)? Social Science and Medicine. 2014;104:56-63.
29. Rowe A, Rowe S, Holloway K, Ivanovska V, Mube L, Lambrechts T. Does shortening the training on Integrated Management of Childhood Illness guidelines reduce its effectiveness? A systematic review. Health Policy and Planning. 2012;27:179-93.
30. Manzi A, Hirschhorn L, Sherr K, Chirwa C, Baynes C, Awoonor-Williams J, et al. Mentorship and coaching support strengthening healthcare systems: lessons learned across the five Population Health Implementation and Training partnership projects in sub-Saharan Africa. BMC Health Services Research. 2017;17.
31. World Health Organisation. Guidelines for follow-up after training in the WHO/UNICEF course on Integrated Management of Childhood Illness for first-level health workers. 1999.
32. Asibon A, Lufesi N, Choudhury A, Olvera S, Molyneux E, Oden M, et al. Using a peer mentorship approach improved the use of neonatal continuous positive airway pressure and related outcomes in Malawi. Acta Paediatrica. 2020;109(4):705-10.
33. Guenther T, Mopiwa G, Nsona H, Qazi S, Makuluni R, Fundani CB, et al. Feasibility of implementing the World Health Organization case management guideline for possible serious bacterial infection among young infants in Ntcheu district, Malawi. PLOS ONE. 2020;15(4):e0229248.
34. McCollum ED, King C, Deula R, Zadutsa B, Mankhambo L, Nambiar B, et al. Pulse oximetry for children with pneumonia treated as outpatients in rural Malawi. Bull World Health Organ. 2016;94(12):893-902.
35. McCambridge J, Witton J, Elbourne D. Systematic review of the Hawthorne effect: New concepts are needed to study research participation effects. Journal of Clinical Epidemiology. 2014;67(3):267-77.

Confidential: For Review Only

Table 1. Health facility and healthcare worker cadre inclusion, by district

**Facilities**	**Total**	**Mchinji**	**Zomba**
Dispensary	7	4	3
Health centre	32	8	24
Rural hospital	3	1	2
CHAM hospital	5	3	2
Total	47	16	31
**Healthcare worker survey respondents**			
Clinical officer	17 (3%)	6 (3%)	11 (3%)
Medical assistant	47 (9%)	15 (8%)	32 (9%)
Nurse/midwife	105 (20%)	32 (17%)	73 (22%)
HSA	190 (36%)	69 (36%)	121 (36%)
Hospital attendant	172 (32%)	71 (37%)	101 (30%)
Total	531	193	338

Confidential: For Review Only

Table 2. *Drug availability and proportion of drugs in date by facility type*

	CHAM hospital (n=5)	Rural hospital (n=3)	Health Centre (n=32)	Dispensary (n=7)
Cotrimoxazole tablet	4 (80%)	3 (100%)	32 (100%)	6 (86%)
Amoxicillin tablets*	5 (100%)	1 (33%)	6 (19%)	1 (14%)
Amoxicillin syrup	4 (80%)	0	20 (63%)	4 (57%)
Lumefantrine-Artemether	5 (100%)	3 (100%)	32 (100%)	6 (86%)
Rectal artesunate	1 (20%)	1 (33%)	19 (59%)	1 (14%)
Oral rehydration salts	5 (100%)	0	16 (50%)	3 (43%)
Salbutamol tablets	5 (100%)	2 (67%)	15 (47%)	3 (43%)
Salbutamol nebuliser solution	4 (80%)	1 (33%)	5 (16%)	7 (100%)
Aminophylline injection**	2 (67%)	1 (100%)	3 (38%)	3 (75%)

**Availability of amoxicillin syrup and/or tables: 5 (100%) of CHAM facilities; 1 (33%) rural hospitals; 22 (69%) health centres; 4 (57%) dispensaries.*

*** Data only collected for Mchinji district (n=16)*

The following drugs were found not to be in date: 20% of ORS at CHAM facilities; 5% of amoxicillin syrup in health centres and 25% at dispensaries; 33% aminophylline at health centres.

Confidential: For Review Only

Table 3. Equipment Availability by facility type and proportion of functional equipment

	CHAM hospital (n=5)		Rural hospital (n=3)		Health Centre (n=32)		Dispensary (n=7)	
	**Available (n, %)**	**Functional (%)**	**Available (n, %)**	**Functional (%)**	**Available (n, %)**	**Functional (%)**	**Available (n, %)**	**Functional (%)**
Thermometer	5 (100%)	100%	3 (100%)	100%	25 (78%)	96%	7 (100%)	71%
Respiratory rate timer	1 (20%)	100%	0	-	6 (19%)	67%	2	100%
MUAC tape	5 (100%)	100%	3 (100%)	100%	32 (100%)	100%	7 (100%)	100%
Weighing scale	5 (100%)	100%	3 (100%)	100%	32 (100%)	100%	7 (100%)	100%
Micro-nebulizer	3 (60%)	100%	1 (33%)	100%	9 (28%)	78%	2	100%
Malaria RDTs	5 (100%)	100%	3 (100%)	100%	31 (97%)	100%	7 (100%)	100%
Pulse oximeter*	3 (60%)	100%	1 (33%)	100%	10 (31%)	70%	0	-

RDT = rapid diagnostic test; MUAC = mid-upper arm circumference

*Pulse oximetry is recommended but not essential according to IMCI guidelines

Confidential: For Review Only

Table 4. IMCI knowledge score description and multivariable linear regression analysis

VARIABLES	Mean score (n=531)				Coefficient (95% CI)	p-value
	N	Mean	Mchinji (n=193)	Zomba (n=338)		
Training						
No training	309	3.69	3.54	3.77	*Ref*	
IMCI training only	137	4.31	4.40	4.26	0.083 (-0.238, 0.405)	0.611
IMCI training + refresher	85	4.40	4.26	4.50	0.417 (0.012, 0.821)	0.043
Cadre						
Clinical Officer	17	4.76	4.67	4.82	*Ref*	
assistant Medical	47	4.57	4.73	4.50	-0.241 (-0.985, 0.503)	0.526
Nurse/midwife	105	4.62	4.63	4.62	-0.149 (-0.834, 0.536)	0.669
HSA	190	4.13	4.11	4.13	-0.820 (-1.500, -0.142)	0.018
Hospital Attendant	172	3.13	3.04	3.19	-1.605 (-2.286, -0.923)	<0.001
Facility type						
Dispensary	40	3.98	4.29	3.81	*Ref*	
Health Centre	378	4.01	3.91	4.04	0.136 (-0.298, 0.571)	0.538
Rural Hospital	113	3.80	3.76	3.92	-0.004 (-0.501, 0.493)	0.987
District						
Mchinji	193	3.87			*Ref*	
Zomba	338	4.01			0.001 (-0.264, 0.266)	0.993

Adjusted R-squared: 0.173

Confidential: For Review Only

Appendix 1: Summary of healthcare worker cadres and their role

**Qualification**	**Definition**
Health Surveillance Assistants	Community level workers with 6 weeks of initial health pre-service training. Generally, provide health promotion and preventive health care through door to door or outreach clinics. Diagnose and treat some illness as part of iCCM in more remote/underserviced areas [1].
Hospital Attendant	Workers who perform routine patient personal care as directed by more senior health personnel. It includes orderlies and nursing aides [2].
Medical Assistants	Two years of clinical training [1] to gain a certificate in clinical medicine. Perform clinical duties (except for surgical procedures) [3].
Clinical Officer	Mid-level practitioners who receive 3-4 years of training and often manage health facilities and fill the role of doctors due to workforce shortages [1, 4]

1. Makwero M. Delivery of primary health care in Malawi. *African Journal of Primary Health Care & Family Medicine*. 2018;10(1)
2. Malawi Health Workforce Observatory. *Human Resources for Health Country Profile Malawi,*. 2010.
3. Muula A. Case for Clinical Officers and Medical Assistants in Malawi. *Croatian Medical Journal*. 2009;50(1):77-78.
4. Jiskoot P. On-the-job training of clinical officers in Malawi. *Malawi Medical Journal*. 2008;20(3):74-77.

Confidential: For Review Only

Appendix 2: Healthcare worker focus group discussion topic guide

1. Current IMCI

- When did you last have IMCI training? Can you describe the training?
- Can you describe what IMCI is?
- Do you think you implement IMCI in your setting?
- Are there any barriers you face in implementing IMCI?
- Are there any benefits you think of implementing IMCI?

2. Current emergency treatment

- Can you give a recent example of child you saw, that you would describe as an emergency?
    - Probe: do others recognise this example, ask for multiple different ones, you can give an example
- Can you describe what you did with this child?
    - Probe: would everyone do the same, what would they do different
- What would you have liked to do in this situation?
- How did you feel about your management of this case?
- How often do you see children like this?
- Do you use any tools or algorithms (guidelines) when assessing emergency cases?
- When managing emergency cases, how do you work? Is it alone, or in a team? Who is part of the team?

3. Current referral procedure

- How often do you refer a child to the hospital? For example, this week how many children has everyone referred?
- Can you describe the process of referral?
    - Probe: documentation, explaining to the caregiver, organising transport
- Do all caregivers complete the referral?
- Are there any barriers you face in referring children?
- Are there any benefits to referring children?

4. ETAT awareness

- How do you decide which order to see patients in your clinic?
- Have you heard of ETAT? Can you describe ETAT?
- Have you ever had training in ETAT? What about other types of triaging?

5. ETAT preparedness

- What steps would need to happen for you implement ETAT?
- Do you have the equipment you need? Can you give examples?
- Do you have the staff you need?
- Are there any barriers to implementing ETAT? And barriers to adequate management of severely ill children?
- Are there any benefits to implementing ETAT in your setting?

Confidential: For Review Only

Appendix 3: Summary of healthcare worker IMCI knowledge scores, with 10 being the maximum score

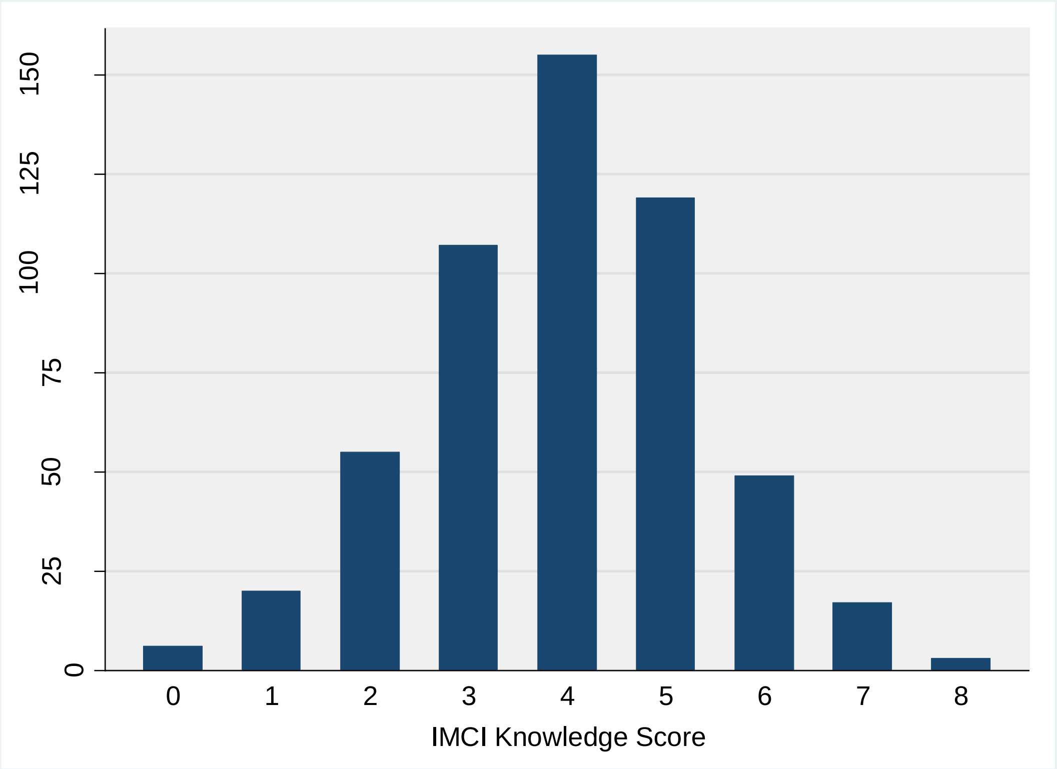

For Review Only

Appendix 4: Healthcare provider IMCI training and knowledge survey

1.1 Have you ever had IMCI training?	Yes / No	If YES, go to 1.4
1.2 Have you ever been given an orientation or briefing on IMCI?	Yes / No	If NO, go to 1.9
1.3 Who gave you this orientation/ briefing?	1. Facility in-charge<br>2. Other facility staff<br>3. DHMT representative<br>4. Other	Go to 1.9
1.4 When was your first training?	_______ year	
1.5 Who provided this first training?	1. MoH/DHMT<br>2. NGO<br>3. Research project<br>4. During school/training<br>5. Other	
1.6 Have you had a refresher training or active mentorship since?	Y / N	If NO, go to 1.9
1.7 When was the refresher?	_______ year	
1.8 Who provided this refresher training?	1. MoH / DHTM<br>2. NGO<br>3. Research project<br>4. During school/training<br>5. Other	Select all that apply
1.9 What is a child's classification if he is 10 months old, has had a cough that lasted two days, has a breathing rate of 46 breaths per minute and chest indrawing?[1]	1. Cough or cold<br>2. Pneumonia<br>3. Severe pneumonia<br>4. Very severe febrile disease<br>5. Don't know	Select one
1.10 What are the four main symptoms for which every sick child should be checked?	1. Malnutrition, cough, vitamin A, ear problems<br>2. Anemia, fever, diarrhea, ear problem<br>3. Cough, diarrhea, malnutrition, ear problem<br>4. Cough, diarrhea, fever, ear problem<br>5. Don't know	Select the best answer
1.11 For each of the following cases, select **Yes** if urgent referral is needed or select **No** if urgent referral is not needed.		
1.11a A 6-month-old boy does not have general danger signs. He is classified with: MASTOIDITIS and NO ANEMIA AND NOT VERY LOW WEIGHT	1. Yes<br>2. No<br>3. Don't know	
1.11b A 7-month-old girl does not have general danger signs. She is classified with: COUGH and DIARRHEA	1. Yes<br>2. No<br>3. Don't know	

Confidential: For Review Only

1.11c A 9-month-old boy is lethargic. He is classified with: DIARRHEA and WITH SEVERE DEHYDRATION	1. Yes<br>2. No<br>3. Don't know	
1.11d A 2-year-old girl does not have general danger signs. She is classified with: DIARRHEA and SEVERE DEHYDRATION and SEVERE MALNUTRITION	1. Yes<br>2. No<br>3. Don't know	
1.12 If a child has any of the four general danger signs, you should urgently refer him to hospital for treatment, These signs are:	1. Unable to drink/feed<br>2. Severe cough<br>3. Convulsions<br>4. Vomiting everything<br>5. Lethargy / unconsciousness<br>6. Bloody stools<br>7. Don't know	Select four from the list
1.13 According to IMCI, a mother of a sick child should be counselled about what topics:	1. Importance of fluids and feeding<br>2. When to return to the clinic immediately<br>3. Her own health<br>4. Immunisation<br>5. When to return for a follow-up visit<br>6. The treatments being given to the child<br>7. Family planning<br>8. Don't know	Select all that apply
1.14 What are two signs that are used to classify severe malnutrition	1. Small arm circumference<br>2. Visible severe wasting<br>3. Oedema of both feet<br>4. Severe dehydration<br>5. Don't know	Select two from the list
1.15 To classify the dehydration status of a child with diarrhoea you will look:	1. At the general condition<br>2. For sunken eyes<br>3. For oedema of both feet<br>4. If the child is drinking eagerly<br>5. For palmar pallor<br>6. For a swollen abdomen<br>7. Don't know	Select all that apply

Confidential: For Review Only

BMJ Paediatrics Open

Integrated Management of Childhood Illnesses (IMCI): A mixed-methods study on implementation, knowledge and resource availability in Malawi

Journal:	*BMJ Paediatrics Open*
Manuscript ID	bmjpo-2021-001044.R2
Article Type:	Original research
Date Submitted by the Author:	09-Apr-2021
Complete List of Authors:	Kilov, Kim; Karolinska Institute, Department of Global Public Health<br>Hildenwall, Helena; Karolinska Institute, Department of Global Public Health<br>Dube, Albert; Malawi Epidemiology and Intervention Research Unit (MEIRU)<br>Zadutsa, Beatiwel; Parent and Child Health Initiative<br>Banda, Lumbani; Parent and Child Health Initiative<br>Langton, Josephine; University of Malawi College of Medicine, Paediatrics<br>Desmond, Nicola; Malawi-Liverpool-Wellcome Trust Programme<br>Lufesi, Norman; Ministry of Health<br>Makwenda, Charles; Parent and Child Health Initiative<br>King, Carina; University College London, Institute for Global Health; Karolinska Institute, Department of Global Public Health
Keywords:	Health services research

SCHOLARONE™
Manuscripts

I, the Submitting Author has the right to grant and does grant on behalf of all authors of the Work (as defined in the below author licence), an exclusive licence and/or a non-exclusive licence for contributions from authors who are: i) UK Crown employees; ii) where BMJ has agreed a CC-BY licence shall apply, and/or iii) in accordance with the terms applicable for US Federal Government officers or employees acting as part of their official duties; on a worldwide, perpetual, irrevocable, royalty-free basis to BMJ Publishing Group Ltd ("BMJ") its licensees and where the relevant Journal is co-owned by BMJ to the co-owners of the Journal, to publish the Work in this journal and any other BMJ products and to exploit all rights, as set out in our licence.

The Submitting Author accepts and understands that any supply made under these terms is made by BMJ to the Submitting Author unless you are acting as an employee on behalf of your employer or a postgraduate student of an affiliated institution which is paying any applicable article publishing charge ("APC") for Open Access articles. Where the Submitting Author wishes to make the Work available on an Open Access basis (and intends to pay the relevant APC), the terms of reuse of such Open Access shall be governed by a Creative Commons licence – details of these licences and which Creative Commons licence will apply to this Work are set out in our licence referred to above.

Other than as permitted in any relevant BMJ Author's Self Archiving Policies, I confirm this Work has not been accepted for publication elsewhere, is not being considered for publication elsewhere and does not duplicate material already published. I confirm all authors consent to publication of this Work and authorise the granting of this licence.

Confidential: For Review Only

Title: Integrated Management of Childhood Illnesses (IMCI): A mixed-methods study on implementation, knowledge and resource availability in Malawi

Authors: Kim Kilov[1], Helena Hildenwall[1,2,3], Albert Dube[4], Beatiwel Zadutsa[5], Lumbani Banda[5], Josephine Langton[6], Nicola Desmond[7], Norman Lufesi[8], Charles Makwenda[5], Carina King[1,9]*

Affiliations:

1. Department of Global Public Health, Karolinksa Institutet, Stockholm, Sweden
2. Astrid Lindgren Children's Hospital, Karolinska University Hospital, Stockholm, Sweden
3. Department of Clinical Science, Intervention and Technology, Karolinska Institutet, Stockholm, Sweden
4. Malawi Epidemiology and Intervention Research Unit (MEIRU), Lilongwe, Malawi
5. Parent and Child Health Initiative, Lilongwe, Malawi
6. Department of Paediatrics, College of Medicine, Blantyre, Malawi
7. Social Science Unit, Malawi-Liverpool-Wellcome Trust Programme, Blantyre, Malawi
8. Acute Respiratory Infections Unit, Ministry of Health, Lilongwe, Malawi
9. Institute for Global Health, University College London, London, UK

***Corresponding author:**

Department of Global Public Health, Karolinska Institutet, Widerströmska huset, Tomtebodavägen 18B, 171 65 Solna, Sweden. Email: carina.king@ki.se

Keywords:

Integrated Management of Childhood Illness; Malawi; Under-five mortality; Health worker; Knowledge; Resources

Word count: 3644

Confidential: For Review Only

Abstract

Background

The introduction of the World Health Organisation's Integrated Management of Childhood Illnesses guidelines (IMCI) in the mid-1990s contributed to global reductions in under-five mortality. However, issues in quality of care have been reported. We aimed to determine resource availability and healthcare worker knowledge of IMCI guidelines in two districts in Malawi.

Methods

We conducted a mixed-method study, including health facility audits to record availability and functionality of essential IMCI equipment and availability of IMCI drugs, healthcare provider survey and focus group discussions (FGDs) with facility staff. The study was conducted between January to April 2019 in Mchinji (central region) and Zomba (southern region) districts. Quantitative data was described using proportions and chi-squared tests; linear regression was conducted to explore factors associated with IMCI knowledge. Qualitative data was analysed using a pragmatic framework approach. Qualitative and quantitative data were analysed and presented separately.

Results

Forty-seven health facilities and 531 healthcare workers were included. Lumefantrine-Artemether and cotrimoxazole were the most available drugs (98% and 96%); while amoxicillin tablets and salbutamol nebuliser solution were the least available (28% and 36%). Respiratory rate timers were the least available piece of equipment, with only 8 (17%) facilities having a functional device. The mean IMCI knowledge score was 3.96 out of 10, and there was a statistically significant association between knowledge and having received refresher training (coeff: 0.42; 95% CI: 0.01-0.82). Four themes were identified in the FGDs: IMCI implementation and practice, barriers to IMCI, benefits of IMCI and sustainability.

Conclusion

We found key gaps in IMCI implementation, however these were not homogenous across facilities, suggesting opportunities to learn from locally adapted IMCI best practices. Improving on-going mentorship, training and supervision should be explored to improve quality of care, and programming which moves away from vertical financing with short term support, to a more holistic approach with embedded sustainability may address the balance of resources for different conditions.

Confidential: For Review Only

Summary Box:

What is known about the subject

- WHO's integrated management of childhood illness guidelines have been important in reducing child deaths in low-resource settings
- However, quality of care issues in IMCI implementation have been noted, including lack of resources, drugs and trained staff
- Training in IMCI is variable, and recommendations for and evidence on the impact of refresher trainings is limited

What this study adds

- In two districts in Malawi, we found key IMCI implementation gaps; these were not homogenous across facilities suggesting opportunities to learn from locally adapted best practice
- Training alone was not independently associated with improved IMCI knowledge, but having had refresher training was.
- Resource gaps were more prominent for respiratory and diarrhoeal case management than malaria, highlighting issues in vertical financing

Confidential: For Review Only

Introduction

Despite a reduction in under five deaths globally from 12.5 million in 1990 to 5.3 million in 2018, progress has been uneven (1). The highest burden is seen in Sub-Saharan Africa, with considerable national and sub-national variation (1). A key strategy in the success to date was the introduction of the WHOs Integrated Management of Childhood Illness (IMCI) guidelines (2). At the time, the major causes of child mortality were pneumonia, malaria, measles, malnutrition and diarrhoea (2). More recently this has shifted to neonatal complications (1), while pneumonia remains the leading infectious cause of mortality in children under-five (3).

IMCI was intended for countries with under-five mortality rates higher than 40 per 1000 live births, and focuses on three components: improving case management skills, strengthening health systems and improving community health practices (4, 5). While IMCI has been partially or fully adopted by 100 countries (6, 7), a survey from 2016 reported only 44 countries were considered to be fully implementing it (2). Further, IMCI coverage was lowest in countries with the highest mortality rates; of the 26 countries to achieve the Millennium Development Goal 4 - reducing under-five mortality by two thirds, 20 had fully implemented IMCI (2).

Key strengths of IMCI are the holistic approach, rational use of medications and improved quality and efficiency of health service provision (2, 7). However, implementation barriers and inconsistencies have been reported. For example, the WHO guidance for training contains 7 modules taught over 11 days, but local adaptations have led to considerable differences, with examples of both shorter and distance learning approaches (8, 9). Currently there is a lack of standardised guidance on the frequency, content, pedagogical approach and expected learning outcomes for refresher courses.

Malawi was an early adopter of IMCI, first implementing it in 2000 (10), and was one of only 10 low-income countries in Sub-Saharan Africa to achieve MDG4 (11). This success has been attributed to proactive policies, scale-up and introduction of vaccinations, coverage of insecticide treated bed nets, IMCI and integrated community case management (10). However, Malawi is not currently on track to achieve the target set out in Sustainable Development Goal 3.2, and faces considerable challenges with an under-resourced health sector with workforce shortages (12). Previous studies have reported suboptimal IMCI implementation for pneumonia, with issues in clinical assessments, quality of diagnosis and antibiotic prescription (13, 14).

Given changes in causes of under-five mortality, varied successes in IMCI implementation and ongoing challenges of under-resourced health systems, it is important to assess if IMCI is effectively supported and implemented. We aimed to describe current IMCI implementation at primary care in

Confidential: For Review Only

Malawi, and determine whether there were sufficient resources and trained staff available to assess and manage children under-five according to these guidelines.

Methods

We conducted a concurrent mixed-methods study, covering all dispensaries, health centres, rural hospitals and Christian Health Association of Malawi (CHAM) hospitals in Zomba and Mchinji districts. Data was collected between January – April 2019. The study included facility audits to assess resource availability, healthcare provider surveys to assess training and knowledge, and focus groups discussions (FGDs) with healthcare providers and facility managers to explore current implementation. Qualitative and quantitative data were analysed and are presented separately.

Setting

Mchinji district is in Malawi's central region, with an under-five population of approximately 90,000 and an under-five mortality rate of 123/1,000 livebirths in the 2015-16 DHS. Zomba is located in the Southern region, with an under-five population of 120,000 and mortality of 54/1,000 livebirths (15, 16). Zomba has a larger urban population and has historically received both higher per capita domestic and external funding (15, 17), while Mchinji has 84% of the population living rurally (15). The health system is made up of three levels (18). Health centres, dispensaries and rural clinics deliver primary care and are linked to community care via Health Surveillance Assistants (HSA) (19). Secondary care is delivered at district hospitals and regional referral hospitals provide tertiary care. A summary of healthcare workers and their roles are presented in Web-Appendix 1. Government care is free, and costs of services delivered by CHAM facilities are subsidised through service level agreements resulting in small out of pocket payments (21).

Sampling

All dispensaries, health centres, rural and CHAM hospitals (i.e., frontline facilities) were eligible to be audited. A convenience sample of staff were recruited for the survey, including staff who interact with paediatric patients and were present at the facility at the time of data collection. Qualitative participants were selected using purposive sampling, aiming to conduct three FGDs in each district. Participants were invited to participate by phone by a member of study staff, with permission from the District Health Management Team and senior HSAs. The groups targeted included: 1. HSAs and attendants at health centres; 2. medical assistants and nurses at health centres; 3. clinical officers and medical assistants at rural hospitals. We invited up to 10 participants per group.

Confidential: For Review Only

Quantitative Data Collection

The audit assessed the number of healthcare workers, availability and functionality of essential IMCI equipment (thermometer, respiratory rate timer, mid-upper arm circumference (MUAC) tape, scale, nebuliser) and availability of IMCI drugs (antibiotics, anti-malarials, oral rehydration solution and salbutamol) used for outpatient case management and pre-referral treatment, and whether they were in date. Data on drugs and equipment used for complex emergency cases are not presented. The structured healthcare worker survey included questions on: cadre, previous IMCI training, refresher courses, years since training and refresher training received and knowledge of the 2014 IMCI guidelines. The knowledge questions were adapted from an IMCI computer-based training course evaluation delivered by USAID in Kenya (20); this was selected due to its short length covering different elements of IMCI knowledge, and the questions should not have been previously seen by participants in our setting.

Data was collected by trained study staff who visited each facility at a pre-arranged time to conduct the audit and surveys. The study staff consisted of clinical officers and monitoring and evaluation officers. The data was collected with support from facility in-charge, pharmacy and health management information staff and included visual inspection of drug stocks to check quantities and expiry dates, equipment functionality and closed questions. Visual inspection by the study staff member was conducted to reduce potential recall and social desirability biases. Data was entered into android tablets using Open Data Kit Collect, with in-built cleaning and skip-pattern rules to promote data quality. The survey was self-completed on an android tablet for healthcare workers who were familiar with smart phone technology. If unfamiliar, the survey was interviewer administered.

Qualitative Data Collection

FGDs were held at healthcare facilities and led by a male Malawian researcher (AD) with experience of qualitative research, supported by a clinical officer to provide IMCI specific knowledge. Discussions were open to be conducted in Chichewa or English, depending on the content and preference of participants. Interviews and discussions were audio-recorded, transcribed and where necessary translated into English. Participants were reimbursed for their travel expenses and provided with refreshments.

Analysis

The audit data was described using proportions, and compared between facility types and districts using Chi-squared tests. Healthcare worker knowledge was summarised into a score with a

Confidential: For Review Only

maximum of 10 points. Mean scores were compared for the following independent variables: IMCI training received, years since training, refresher training received, years since refresher training, cadre, facility type and district. Multivariable linear regression analysis was conducted to assess the association between IMCI knowledge scores and training. The following confounders were considered: district (given different funding levels), facility type, health worker qualification and refresher course. Quantitative analysis was performed using Stata IC 16.0.

FGDs were analysed using a pragmatic framework approach, with pre-defined themes based on the topic guide (21) (Appendix 2). Emergent themes were coded during the analysis. Discrepancies were agreed through discussion, and the interpretation and conclusions shared and discussed with the wider study team. Coding was done by HH, with a sub-set double coded by CK. These codes were then discussed, refined and organised into themes, which were checked by AD for consistency with his interpretation and the local context.

Patient and Public Involvement

Patients and public were not involved in the design or execution of the study. Prior to starting, the protocol was presented to both District Health Management and District Executive Committees (which include local government and civil society representation) in both Zomba and Mchinji. Minor edits to the study plan were instituted following feedback and questions raised in these meetings.

Results

The total 47 health facilities were audited, with 16 in Mchinji and 31 in Zomba (**Table 1**), and 44% (n=531/1197) of clinical staff employed across these facilities were surveyed. All six planned FGDs were completed, with three in each district.

Facility Audits

Availability of seven IMCI drugs was assessed (**Table 2**). Lumefantrine-Artemether and cotrimoxazole were the most available (98% and 96%); while amoxicillin tablets and salbutamol nebuliser solution were the least available (28% and 36%). When available, the majority of drugs were found to be in date. Respiratory rate timers were the least available piece of equipment, with only 8 (17%) facilities across both districts having a functional device. This was followed by pulse oximeters (30%) and micro-nebulisers (32%, **Table 3**). MUAC tapes, scales and mRDTs were almost universally available. Health centres had the highest proportion of non-functional equipment.

Healthcare worker survey

Confidential: For Review Only

Overall 42% (n=222/531) of the survey respondents reported having IMCI training, with no difference by district (p-value=0.900). Of these, 38% had also received refresher training. The most common group to report training were HSAs (78%), while clinical officers, medical assistants and nurses all reported similar levels (45%), and only one attendant reported training. Training was most frequently received from NGOs (50%) followed by Ministry of Health (36%) and during qualification training (14%); refresher trainings were delivered by NGOs (64%) and Ministry of Health (36%). The median time since initial training was 7 years (95% confidence interval: 6, 8; range: 0 - 21).

The mean knowledge score, of a possible 10 points, was 3.96 (95% confidence interval: 3.83, 4.08; range: 0 – 8), with 65% scoring less than 5 (Web Appendix 3). Questions on referral decision using clinical scenarios had both the most correct responses (84% - severe dehydration with another sign of severity), and most incorrect (13% - severe dehydration without any other urgent signs). The multivariable regression found a statistically significant association between IMCI knowledge score and having received refresher training (coeff: 0.42; 95% CI: 0.01-0.82) - **Table 4**. However, training alone was not significantly associated with IMCI knowledge. Being a HSA or hospital attendant were both associated with a lower score compared to clinical officers (coeff: -0.82; 95% CI: -1.50, -0.14 and coeff: -1.61; 95% CI: -2.29, -0.92). Amongst those with training, there was a weak but statistically significant positive correlation between knowledge and years since training (correlation coeff: 0.213; p-value: 0.001).

*Focus group discussions***:**

The qualitative data are presented under the following themes: IMCI implementation and practice, barriers to IMCI effectiveness, benefits of IMCI and sustainability.

1. IMCI implementation and practice

This theme includes healthcare workers understanding of IMCI, real-world adaptations, self-perceived quality of implementation, and training received. Overall IMCI was described as a system of assessment, a holistic approach to care for children and as a way to classify illness severity. Across the discussions it was apparent that there were inconsistencies in the implementation of IMCI, but groups generally described a process of weighing, initial clinical assessment, tests and then assigning a diagnosis. One group from Mchinji specifically talked of how a new electronic patient record system had resulted in more structure.

Healthcare workers discussed doing their best to deliver IMCI, in spite of challenges. Although also acknowledged that they do not always follow the guidelines, whether this was intentional was not clear:

Confidential: For Review Only

"I guess some people are not using the IMCI approach so it is hard to assess properly. Because they are straight from school sometimes you need updates in order to know when everything is working properly and to give an alarm to the people who are using it" (Zomba, FGD 1)

In the groups, there was a mix between those who reported training and those who hadn't received any. Participants in one group described how staff had also received different types of training, depending on who delivered it:

"There were some people who were trained by the Ministry of health, while others were retained by PSI [an international NGO…] it was a week-long training. But those who were trained by other organisations, the training lasted for three weeks." (Zomba, FGD 2)

2. Barriers to IMCI effectiveness

Three main barriers were discussed: community barriers, lack of adequate resources and lack of trained staff capacity. The issue of caregivers presenting late was a key example raised by several groups as a barrier to effective IMCI implementation. This resulted in tensions around communication and trust between caregivers and providers, and participants were aware of the compounding effects of high patient loads, stock-outs and not having 24-hour staffing.

"When we ask her the time the child started getting sick she tells a lie by saying they started getting ill today. If we argue her response, you find that sometimes neighbours will confront her by saying the child started suffering sometime back only that she was busy running her business. That's the challenge we encounter sometimes." (Zomba, FDG 3)

While the presence of community structures to support the facilities were described, they noted that they did not always work together effectively. In terms of resources, this was raised as a major challenge for some, but not others who described well-functioning supply chain management for essential medications, such as antibiotics. Various scenarios of lacking guidelines, medications, diagnostics and equipment were given to highlight how these limit the ability to provide care for children

"Sometimes you can find that the child has high fever and there is a need for the child to be given [anti-malarials], but before that you have to confirm with a malaria test, and if you don't have the test kit it becomes a big challenge"(Zomba, FDG 2)

Confidential: For Review Only

> "The guidelines are not there. Even if you are not trained you can use the guidelines to tell you what to do, but the guidelines are not there" (Zomba, FDG 1)

Multiple dimensions of staffing were raised, from high workloads restricting the amount of time that could be spent conducting an IMCI assessment, not all staff being trained, and lacking supportive supervision. One discussion with providers from a CHAM hospital however noted that they had no problems with staffing unless there were absences due to events such as funerals.

3. Benefits of IMCI

Benefits of IMCI included better clinical outcomes and better ways of working. Generally, many of the participants acknowledged that IMCI results in improved outcomes for children, and that this also translates into care and care-seeking practices amongst the community.

> "It helps mothers to have additional knowledge of caring [for their] children, through what she is advised at hospital by health workers" (Zomba, FDG 2)

IMCI was described as reducing the workload for hospitals, as frontline facility staff were able to treat conditions such as malaria. The structured and holistic approach was valued by participants in both districts, for example:

> "Yes, it provides a holistic approach to childhood illness, it gives a knowledge on how to treat under-five children. It is a lifesaving system." (Mchinji, FDG 1)

4. IMCI Sustainability

This theme addresses the long term sustainability of IMCI implementation in frontline facilities, with both end-user ownership and sustaining capacity discussed. Notably, this topic was only raised by healthcare providers in Mchinji and included both challenges and concrete recommendations. A key challenge was around the transfer of trained staff, especially when not all staff are sent for IMCI training. Around who attends training, there were concerns around transparency of who is sent for training, which can compound the issue.

> "Yes, it is a problem, for instance someone has gone for IMCI training and next month has been transferred, that means we will have a problem with IMCI because that person didn't finish sharing the information." (Mchinji, FDG 2)

https://mc.manuscriptcentral.com/bmjpo

Confidential: For Review Only

To promote sustainable programming there was a request for healthcare workers to be involved in the development process, and ensuring motivation and follow-up was sustained. NGO instituted programmes were specifically called out for this practice:

> "Another problem, it's you NGO people, you don't follow up the programmes" (Mchinji, FDG 3).

Discussion

In this study we explored how IMCI is currently being implemented within two districts in Malawi. We found that there were mixed experiences with both implementation and access to essential supplies, notably with important differences between target infections. In terms of healthcare workers, we found refresher training to be more important in terms of IMCI knowledge than cadre of the healthcare worker and having training alone. These findings pose important topics for further exploration and discussion.

The ability to diagnose and treat respiratory conditions in general, and particularly pneumonia, in frontline facilities was poor. This is especially notable when compared to the almost universal access to malaria RDTs and treatment. ORS, as a treatment for diarrhoeal diseases, was also not readily available at government facilities. The IMCI guidelines recommend seven essential drugs for treating malaria, pneumonia, diarrhoea and ear infections. Several facilities lacked the necessary equipment and first-line treatments to diagnose and treat pneumonia, with the exception of cotrimoxazole, which is a first-line treatment for respiratory infections in children with HIV.

Donor funding for child health has steadily increased since the MDGs were introduced in 2000 and this has coincided with increased policies for malaria prevention and treatment in Malawi (10). This is reflected in well-funded malaria programmes in Malawi (Malawi Malaria Communication Strategy 2015-2020 (22)), and in the global funding landscape where malaria has been prioritised through the Global Fund, Bill and Melinda Gates Foundation and USAID (23, 24). This contrasts to pneumonia and diarrhoea, which despite having high burdens, do not receive the same level of strategic funding by large-scale donors. In particular, pneumonia has generally received lower research funding comparative to its global mortality burden (25). This vertical programming focus is often disease specific and detracts from the holistic approach to assessing childhood illness, which IMCI promotes. Further exploration of factors which promote sustainability at different levels of the health system could reveal important insights for future programming.

https://mc.manuscriptcentral.com/bmjpo

Confidential: For Review Only

Resource availability impacts healthcare workers' abilities to adequately perform IMCI assessments and treat according to guidelines. This was apparent from the FGDs with providers, and consistent with previous studies from similar settings, which found better health worker performance in facilities with availability of all essential IMCI equipment (26). Given that some health facilities were better resourced than others, it would be useful to understand different funding allocation, stock management and personnel structures, to ascertain best practices.

The assessment of health care worker IMCI knowledge found more than half of healthcare workers scored under 50%, and those with higher qualifications did not necessarily score better. Completion of both IMCI training and a refresher course was associated with higher IMCI knowledge. This is in line with other studies on IMCI performance, where healthcare workers who had received IMCI training without a refresher course, performed similarly to those without training (27). It is important to note that while training doesn't necessarily translate into knowledge, knowledge does not always result in adherence to IMCI guidelines and improved practice. A previous study found that those with more experience felt that following the guidelines was not necessary (27). Time constraints and a high work load have also been reported to reduce adherence to IMCI guidelines (28).

We found a weak positive relationship between time since training and knowledge, which may reflect the importance of long-term clinical experience rather than training alone. A systematic review by Rowe et al. (2012) reported that healthcare workers performed better with IMCI training than without, but amongst those trained, over a third of ill children were not treated according to IMCI guidelines (29). Traditional training programs do not necessarily translate to adequate knowledge and skills without ongoing support, and findings from five sub-Saharan African countries found coaching and mentorship improved IMCI service delivery (30). The WHO recommends following up four weeks after the initial IMCI training course to help reinforce the skills learned, and continued supervision (31). There are examples of successful mentorship within child health (32, 33), and community case management in Malawi. However, as lacking supervision and mentorship was raised by healthcare workers in the FGDs, work is needed on effective adoption at frontline facilities.

The qualitative data highlighted a key issue around programme sustainability, and the need for local ownership. Training and refresher courses were often provided by NGOs (through external funding), not provided to all staff, and the decision for who is trained was not thought to be transparent. Given the frequent transfer of healthcare workers across different facilities, this results in an unequal distribution of workers with IMCI knowledge. It is therefore important that sustainability

https://mc.manuscriptcentral.com/bmjpo

Confidential: For Review Only

and handover plans are established by NGOs and research groups, which are agreed up-front with District Health Management Teams.

This study had three key limitations, firstly, that the two districts were selected purposively, and our findings may not be representative to other districts. For example, Mchinji has had multiple research projects in the last decade which have included IMCI refresher trainings and health system strengthening (e.g., McCollum et al. 2016 (34)). In particular, we did not include any district from the northern region. Secondly, the healthcare workers in the survey were those present on the day, and therefore may not cover the full range of providers (e.g., those who predominantly focus on outreach services). The frequency of healthcare worker cadres generally reflected the health workforce composition in Malawi, where HSAs constitute one third of the workforce and few clinical officers (19). The higher proportion of workers from Zomba is also expected given it has a larger population compared to Mchinji, and has traditionally received higher healthcare funding (17). Finally, assessment of practice was not performed, with direct observations of health worker performance posing several ethical and methodological challenges (e.g. the Hawthorne effect (35)). We therefore cannot comment on quality of IMCI care provision.

Malawi's early adoption and scaled-up implementation of IMCI has likely played a key role in the successes made in reducing under-five mortality, but further progress is needed to reach the SDG 3.2 goal. While we found expected challenges in staffing and resources, there are several opportunities for improvement and further research. Firstly, determining which combination of supervision, mentorship and re-training is most cost-effective within the specific context of frontline facilities, to ensure improved knowledge and subsequent quality of care. Secondly, to explore locally adapted IMCI best practices from those facilities which had good supply chain management, positive attitudes to staff transfers and sufficient trained staff capacity. Finally, supporting programming to move away from vertical financing with short term support, to the more holistic approach which IMCI promotes and considers strategies for embedded sustainability.

Confidential: For Review Only

Acknowledgements

We would like to thank the District Health Office in Mchinji and Zomba for supporting this project, and all the healthcare providers, facility managers, technicians and support start for sharing their time and knowledge. We acknowledge Christopher Kampinga, Tarcizious Phiri, Harry Liyaya (PACHI, Malawi) and Clifford Katumbi (College of Medicine, Malawi) for collecting the audit and survey data.

Funding

This study was funded by a project grant the Swedish Research Council 2017-05579. The funder did not play any role in the design, analysis or interpretation of the data. The analysis and write up was undertaken as part of an MSc student project (the student received a Global Grant Scholarship through Rotary International to undertake a graduate study program).

Conflict of Interest

None declared.

Author Contributions

The study was conceived by HH and CK and study protocols and data collection tools designed together with BZ, CM, LB, ND and AD. Quantitative data collection was overseen by BZ, LB and CM and qualitative data collection was conducted by AD, with support from BZ. Quantitative analysis was conducted by KK with input from CK and HH. Qualitative analysis was conducted by CK and HH, with input from AD and KK. The manuscript was drafted by KK, with significant contributions from CK, HH and AD. All authors read and approved the final manuscript.

Confidential: For Review Only

References

1. The United Nations Children's Fund WHO, World Bank Group, United Nations. Levels and Trends in Child Mortality. Report 2019. New York: UNICEF; 2019.
2. World Health Organisation. Integrated Management of Childhood Illness global survey report. Geneva: World Health Organisation; 2017.
3. GBD 2019 Diseases and Injuries Collaborators. Global burden of 369 diseases and injuries, 1990–2019: a systematic analysis for the Global Burden of Disease Study 2019. The Lancet. 2020.
4. World Health Organisation. Integrated Management of Childhood Illness (IMCI) 2019 [Available from: https://www.who.int/maternal_child_adolescent/topics/child/imci/en/.
5. World Health Organisation. IMIC: Improving Child Health. Geneva: WHO; 1999.
6. Jacobs M, Merson M. Introductory commentary: a strategic review of options for building on lessons learnt from IMCI and iCCM. British Medical Journal. 2018;362.
7. Boschi-Pinto C, Labadie G, Ramachandandran Dilip T, Oliphant N, Dalgish S, Aboubaker S, et al. Global implementation survey of Integrated Management of Childhood Illness (IMCI): 20 years on. BMJ Open. 2018;8.
8. Goga AE, Muhe LM, Forsyth K, Chopra M, Aboubaker S, Martines J, et al. Results of a multi-country exploratory survey of approaches and methods for IMCI case management training. Health Research Policy and Systems. 2009;7(1):18.
9. Nguyen DTK, Leung KK, McIntyre L, Ghali WA, Sauve R. Does Integrated Management of Childhood Illness (IMCI) Training Improve the Skills of Health Workers? A Systematic Review and Meta-Analysis. PLOS ONE. 2013;8(6):e66030.
10. Kanyuka M, Ndawala J, Mleme T, Chisesa L, Makwemba M, Amouzou A, et al. Malawi and Millennium Development Goal 4: a Countdown to 2015 country case study. The Lancet Global Health. 2016;4:e201-14.
11. Molyneux M, Molyneux E. Reaching Millennium Development 4. The Lancet Global Health. 2016;4(3):PE146-E7.
12. Wemos Foundation. Country Report Malawi. Minding the gap: Who is paying the health workers? Mind the funding gap:. Amsterdam; 2018.
13. Johansson E, Humphreys N, Carvajal-Aquirre L, Amouzou A, Hildenwall H. Determinants of Integrated Management of Childhood Illness (IMCI) non-severe pneumonia classification and care in Malawi health Facilities: Analysis of a national facility census,. Journal of Global Health. 2017;7(2).
14. Bjornstad E, Preidis G, Lufesi N, Olson D, Kamthunzi P, Hosseinipour M, et al. Determining the quality of IMCI pneumonia care in Malawian children. Paediatric International Child Health. 2014;34(1):29-36.
15. National Statistical Office. Malawi Population and Housing Census. Government of Malawi; 2019.
16. National Statistical Office/Malawi, ICF. Malawi Demographic and Health Survey 2015-16. Zomba, Malawi: National Statistical Office and ICF; 2017.
17. Borghi J, Munthali S, Million L, Martinez-Alvarez M. Health financing at district level in Malawi: an analysis of the distribution of funds at two points in time. Health Policy and Planning. 2018;33(1):59-69.
18. Makwero M. Delivery of primary health care in Malawi. African Journal of Primary Health Care & Family Medicine. 2018;10(1).
19. Angwenyi VA, C. Kondowe, K., Zulu Mutchiyeni JK, MCriel, B. Lazarus, J.V., Quinlan T, Bunders-Aelen J. Moving to a strong(er) community health system: analysing the role of community health volunteers in the new national community health strategy in Malawi. BMJ Global Health. 2018;3:1-10.
20. Quality Assurance Project. Evaluation of an IMCI Computer-based Training Course in Kenya. *Operations Research Results.* . Bethesda, MD: U.S. Agency for International Development (USAID) 2006.

Confidential: For Review Only

21. Gale NK, et al. Using the framework method for the analysis of qualitative data in multi-disciplinary health research. BMC Medical Research Methodology. 2013;13(1):117.
22. Ministry of Health Malawi. Malawi Malaria Communication Strategy 2015-2020. 2015.
23. USAID. President's Malaria Initiative Malawi Abbreviated Malaria Operational Plan FY 2019.
24. The Global Fund. Results Report. Geneva, Switzerland; 2019.
25. Head M, Fitchett J, Nageshwaran V, Kumari N, Hayward A, Atun R. Research Investments in Global Health: A Systematic Analysis of UK Infectious Disease Research Funding and Global health Metrics, 1997-2013. EBioMedicine. 2016;3:180-90.
26. Pariyo G, Gouws E, Bryce J, Burnham G, Uganda IMCI impact study team. Improving facility-based care for sick children in Uganda: training is not enough. Health Policy and Planning. 2005;20.
27. World Health Organisation. Global Strategy on Human Resources for Health: Workforce 2030 Geneva, Switzerland; 2016.
28. Lange S, Mwisongo A, Mæstad O. Why don't clinicians adhere more consistently to guidelines for the Integrated Management of Childhood Illness (IMCI)? Social Science and Medicine. 2014;104:56-63.
29. Rowe A, Rowe S, Holloway K, Ivanovska V, Mube L, Lambrechts T. Does shortening the training on Integrated Management of Childhood Illness guidelines reduce its effectiveness? A systematic review. Health Policy and Planning. 2012;27:179-93.
30. Manzi A, Hirschhorn L, Sherr K, Chirwa C, Baynes C, Awoonor-Williams J, et al. Mentorship and coaching support strengthening healthcare systems: lessons learned across the five Population Health Implementation and Training partnership projects in sub-Saharan Africa. BMC Health Services Research. 2017;17.
31. World Health Organisation. Guidelines for follow-up after training in the WHO/UNICEF course on Integrated Management of Childhood Illness for first-level health workers. 1999.
32. Asibon A, Lufesi N, Choudhury A, Olvera S, Molyneux E, Oden M, et al. Using a peer mentorship approach improved the use of neonatal continuous positive airway pressure and related outcomes in Malawi. Acta Paediatrica. 2020;109(4):705-10.
33. Guenther T, Mopiwa G, Nsona H, Qazi S, Makuluni R, Fundani CB, et al. Feasibility of implementing the World Health Organization case management guideline for possible serious bacterial infection among young infants in Ntcheu district, Malawi. PLOS ONE. 2020;15(4):e0229248.
34. McCollum ED, King C, Deula R, Zadutsa B, Mankhambo L, Nambiar B, et al. Pulse oximetry for children with pneumonia treated as outpatients in rural Malawi. Bull World Health Organ. 2016;94(12):893-902.
35. McCambridge J, Witton J, Elbourne D. Systematic review of the Hawthorne effect: New concepts are needed to study research participation effects. Journal of Clinical Epidemiology. 2014;67(3):267-77.

Confidential: For Review Only

Table 1. Health facility and healthcare worker cadre inclusion, by district

**Facilities**	**Total**	**Mchinji**	**Zomba**
Dispensary	7	4	3
Health centre	32	8	24
Rural hospital	3	1	2
CHAM hospital	5	3	2
Total	47	16	31
**Healthcare worker survey respondents**			
Clinical officer	17 (3%)	6 (3%)	11 (3%)
Medical assistant	47 (9%)	15 (8%)	32 (9%)
Nurse/midwife	105 (20%)	32 (17%)	73 (22%)
HSA	190 (36%)	69 (36%)	121 (36%)
Hospital attendant	172 (32%)	71 (37%)	101 (30%)
Total	531	193	338

Confidential: For Review Only

Table 2. *Drug availability and proportion of drugs in date by facility type*

	CHAM hospital (n=5)	Rural hospital (n=3)	Health Centre (n=32)	Dispensary (n=7)
Cotrimoxazole tablet	4 (80%)	3 (100%)	32 (100%)	6 (86%)
Amoxicillin tablets*	5 (100%)	1 (33%)	6 (19%)	1 (14%)
Amoxicillin syrup	4 (80%)	0	20 (63%)	4 (57%)
Lumefantrine-Artemether	5 (100%)	3 (100%)	32 (100%)	6 (86%)
Rectal artesunate	1 (20%)	1 (33%)	19 (59%)	1 (14%)
Oral rehydration salts	5 (100%)	0	16 (50%)	3 (43%)
Salbutamol tablets	5 (100%)	2 (67%)	15 (47%)	3 (43%)
Salbutamol nebuliser solution	4 (80%)	1 (33%)	5 (16%)	7 (100%)
Aminophylline injection**	2 (67%)	1 (100%)	3 (38%)	3 (75%)

**Availability of amoxicillin syrup and/or tables: 5 (100%) of CHAM facilities; 1 (33%) rural hospitals; 22 (69%) health centres; 4 (57%) dispensaries.*

*** Data only collected for Mchinji district (n=16)*

The following drugs were found not to be in date: 20% of ORS at CHAM facilities; 5% of amoxicillin syrup in health centres and 25% at dispensaries; 33% aminophylline at health centres.

Confidential: For Review Only

Table 3. Equipment Availability by facility type and proportion of functional equipment

	**CHAM hospital (n=5)**		**Rural hospital (n=3)**		**Health Centre (n=32)**		**Dispensary (n=7)**	
	**Available (n, %)**	**Functional (%)**	**Available (n, %)**	**Functional (%)**	**Available (n, %)**	**Functional (%)**	**Available (n, %)**	**Functional (%)**
Thermometer	5 (100%)	100%	3 (100%)	100%	25 (78%)	96%	7 (100%)	71%
Respiratory rate timer	1 (20%)	100%	0	-	6 (19%)	67%	2	100%
MUAC tape	5 (100%)	100%	3 (100%)	100%	32 (100%)	100%	7 (100%)	100%
Weighing scale	5 (100%)	100%	3 (100%)	100%	32 (100%)	100%	7 (100%)	100%
Micro-nebulizer	3 (60%)	100%	1 (33%)	100%	9 (28%)	78%	2	100%
Malaria RDTs	5 (100%)	100%	3 (100%)	100%	31 (97%)	100%	7 (100%)	100%
Pulse oximeter*	3 (60%)	100%	1 (33%)	100%	10 (31%)	70%	0	-

RDT = rapid diagnostic test; MUAC = mid-upper arm circumference

*Pulse oximetry is recommended but not essential according to IMCI guidelines

Confidential: For Review Only

Table 4. IMCI knowledge score description and multivariable linear regression analysis

**VARIABLES**	**Mean score (n=531)**				**Coefficient (95% CI)**	**p-value**
	N	Mean	Mchinji (n=193)	Zomba (n=338)		
Training						
No training	309	3.69	3.54	3.77	*Ref*	
IMCI training only	137	4.31	4.40	4.26	0.083 (-0.238, 0.405)	0.611
IMCI training + refresher	85	4.40	4.26	4.50	0.417 (0.012, 0.821)	0.043
Cadre						
Clinical Officer	17	4.76	4.67	4.82	*Ref*	
assistant Medical	47	4.57	4.73	4.50	-0.241 (-0.985, 0.503)	0.526
Nurse/midwife	105	4.62	4.63	4.62	-0.149 (-0.834, 0.536)	0.669
HSA	190	4.13	4.11	4.13	-0.820 (-1.500, -0.142)	0.018
Hospital Attendant	172	3.13	3.04	3.19	-1.605 (-2.286, -0.923)	<0.001
Facility type						
Dispensary	40	3.98	4.29	3.81	*Ref*	
Health Centre	378	4.01	3.91	4.04	0.136 (-0.298, 0.571)	0.538
Rural Hospital	113	3.80	3.76	3.92	-0.004 (-0.501, 0.493)	0.987
District						
Mchinji	193	3.87			*Ref*	
Zomba	338	4.01			0.001 (-0.264, 0.266)	0.993

Adjusted R-squared: 0.173

Confidential: For Review Only

Title: Integrated Management of Childhood Illnesses (IMCI): A mixed-methods study on implementation, knowledge and resource availability in Malawi

Authors: Kim Kilov[1], Helena Hildenwall[1,2,3], Albert Dube[4], Beatiwel Zadutsa[5], Lumbani Banda[5], Josephine Langton[6], Nicola Desmond[7], Norman Lufesi[8], Charles Makwenda[5], Carina King[1,9]*

Affiliations:

1. Department of Global Public Health, Karolinksa Institutet, Stockholm, Sweden
2. Astrid Lindgren Children's Hospital, Karolinska University Hospital, Stockholm, Sweden
3. Department of Clinical Science, Intervention and Technology, Karolinska Institutet, Stockholm, Sweden
4. Malawi Epidemiology and Intervention Research Unit (MEIRU), Lilongwe, Malawi
5. Parent and Child Health Initiative, Lilongwe, Malawi
6. Department of Paediatrics, College of Medicine, Blantyre, Malawi
7. Social Science Unit, Malawi-Liverpool-Wellcome Trust Programme, Blantyre, Malawi
8. Acute Respiratory Infections Unit, Ministry of Health, Lilongwe, Malawi
9. Institute for Global Health, University College London, London, UK

***Corresponding author:**

Department of Global Public Health, Karolinska Institutet, Widerströmska huset, Tomtebodavägen 18B, 171 65 Solna, Sweden. Email: carina.king@ki.se

Keywords:

Integrated Management of Childhood Illness; Malawi; Under-five mortality; Health worker; Knowledge; Resources

Word count: 3644

Confidential: For Review Only

Abstract

Background

The introduction of the World Health Organisation's Integrated Management of Childhood Illnesses guidelines (IMCI) in the mid-1990s contributed to global reductions in under-five mortality. However, issues in quality of care have been reported. We aimed to determine resource availability and healthcare worker knowledge of IMCI guidelines in two districts in Malawian districts.

Methods

We conducted a mixed-method study, including health facility audits to record availability and functionality of essential IMCI equipment and availability of IMCI drugs, healthcare provider survey and focus group discussions (FGDs) with facility staff. The study was conducted between January to April 2019 in Mchinji (central region) and Zomba (southern region) districts. Quantitative data was described using proportions and chi-squared tests; linear regression was conducted to explore factors associated with IMCI knowledge. Qualitative data was analysed using a pragmatic framework approach. Qualitative and quantitative data were analysed and presented separately.

Results

Forty-seven health facilities and 531 healthcare workers were included. Lumefantrine-Artemether and cotrimoxazole were the most available drugs (98% and 96%); while amoxicillin tablets and salbutamol nebuliser solution were the least available (28% and 36%). Respiratory rate timers were the least available piece of equipment, with only 8 (17%) facilities having a functional device. The mean IMCI knowledge score was 3.96 out of 10, and there was a statistically significant association between knowledge and having received refresher training (coeff: 0.42; 95% CI: 0.01-0.82). Four themes were identified in the FGDs: IMCI implementation and practice, barriers to IMCI, benefits of IMCI and sustainability.

Conclusion

We found key gaps in IMCI implementation, however these were not homogenous across facilities, suggesting opportunities to learn from locally adapted IMCI best practices. Improving on-going mentorship, training and supervision should be explored to improve quality of care, and programming which moves away from vertical financing with short term support, to a more holistic approach with embedded sustainability may address the balance of resources for different conditions.

Confidential: For Review Only

Summary Box:

What is known about the subject

- WHO's integrated management of childhood illness guidelines have been important in reducing child deaths in low-resource settings
- However, quality of care issues in IMCI implementation have been noted, including lack of resources, drugs and trained staff
- Training in IMCI is variable, and recommendations for and evidence on the impact of refresher trainings is limited

What this study adds

- In two districts in Malawi~~an district~~, we found key IMCI implementation gaps; these were not homogenous across facilities suggesting opportunities to learn from locally adapted best practice
- Training alone was not independently associated with improved IMCI knowledge, but having had refresher training was.
- Resource gaps were more prominent for respiratory and diarrhoeal case management than malaria, highlighting issues in vertical financing

Confidential: For Review Only

Introduction

Despite a reduction in under five deaths globally from 12.5 million in 1990 to 5.3 million in 2018, progress has been uneven (1). The highest burden is seen in Sub-Saharan Africa, with considerable national and sub-national variation (1). A key strategy in the success to date was the introduction of the WHOs Integrated Management of Childhood Illness (IMCI) guidelines (2). At the time, the major causes of child mortality were pneumonia, malaria, measles, malnutrition and diarrhoea (2). More recently this has shifted to neonatal complications (1), while pneumonia remains the leading infectious cause of mortality in children under-five (3).

IMCI was intended for countries with under-five mortality rates higher than 40 per 1000 live births, and focuses on three components: improving case management skills, strengthening health systems and improving community health practices (4, 5). While IMCI has been partially or fully adopted by 100 countries (6, 7), a survey from 2016 reported only 44 countries were considered to be fully implementing it (2). Further, IMCI coverage was lowest in countries with the highest mortality rates; of the 26 countries to achieve the Millennium Development Goal 4 - reducing under-five mortality by two thirds, 20 had fully implemented IMCI (2).

Key strengths of IMCI are the holistic approach, rational use of medications and improved quality and efficiency of health service provision (2, 7). However, implementation barriers and inconsistencies have been reported. For example, the WHO guidance for training contains 7 modules taught over 11 days, but local adaptations have led to considerable differences, with examples of both shorter and distance learning approaches (8, 9). Currently there is a lack of standardised guidance on the frequency, content, pedagogical approach and expected learning outcomes for refresher courses.

Malawi was an early adopter of IMCI, first implementing it in 2000 (10), and was one of only 10 low-income countries in Sub-Saharan Africa to achieve MDG4 (11). This success has been attributed to proactive policies, scale-up and introduction of vaccinations, coverage of insecticide treated bed nets, IMCI and integrated community case management (10). However, Malawi is not currently on track to achieve the target set out in Sustainable Development Goal 3.2, and faces considerable challenges with an under-resourced health sector with workforce shortages (12). Previous studies have reported suboptimal IMCI implementation for pneumonia, with issues in clinical assessments, quality of diagnosis and antibiotic prescription (13, 14).

Given changes in causes of under-five mortality, varied successes in IMCI implementation and ongoing challenges of under-resourced health systems, it is important to assess if IMCI is effectively supported and implemented. We aimed to describe current IMCI implementation at primary care in

Confidential: For Review Only

Malawi, and determine whether there were sufficient resources and trained staff available to assess and manage children under-five according to these guidelines.

Methods

We conducted a concurrent mixed-methods study, covering all dispensaries, health centres, rural hospitals and Christian Health Association of Malawi (CHAM) hospitals in Zomba and Mchinji districts. Data was collected between January – April 2019. The study included facility audits to assess resource availability, healthcare provider surveys to assess training and knowledge, and focus groups discussions (FGDs) with healthcare providers and facility managers to explore current implementation. Qualitative and quantitative data were analysed and are presented separately.

Setting

Mchinji district is in Malawi's central region, with an under-five population of approximately 90,000 and an under-five mortality rate of 123/1,000 livebirths in the 2015-16 DHS. Zomba is located in the Southern region, with an under-five population of 120,000 and mortality of 54/1,000 livebirths (15, 16). Zomba has a larger urban population and has historically received both higher per capita domestic and external funding (15, 17), while Mchinji has 84% of the population living rurally (15). The health system is made up of three levels (18). Health centres, dispensaries and rural clinics deliver primary care and are linked to community care via Health Surveillance Assistants (HSA) (19). Secondary care is delivered at district hospitals and regional referral hospitals provide tertiary care. A summary of healthcare workers and their roles are presented in Web-Appendix 1. Government care is free, and costs of services delivered by CHAM facilities are subsidised through service level agreements resulting in small out of pocket payments (21).

Sampling

All dispensaries, health centres, rural and CHAM hospitals (i.e., frontline facilities) were eligible to be audited. A convenience sample of staff were recruited for the survey, including staff who interact with paediatric patients and were present at the facility at the time of data collection. Qualitative participants were selected using purposive sampling, aiming to conduct three FGDs in each district. Participants were invited to participate by phone by a member of study staff, with permission from the District Health Management Team and senior HSAs. The groups targeted included: 1. HSAs and attendants at health centres; 2. medical assistants and nurses at health centres; 3. clinical officers and medical assistants at rural hospitals. We invited up to 10 participants per group.

https://mc.manuscriptcentral.com/bmjpo

Confidential: For Review Only

Quantitative Data Collection

The audit assessed the number of healthcare workers, availability and functionality of essential IMCI equipment (thermometer, respiratory rate timer, mid-upper arm circumference (MUAC) tape, scale, nebuliser) and availability of IMCI drugs (antibiotics, anti-malarials, oral rehydration solution and salbutamol) used for outpatient case management and pre-referral treatment, and whether they were in date. Data on drugs and equipment used for complex emergency cases are not presented. The structured healthcare worker survey included questions on: cadre, previous IMCI training, refresher courses, years since training and refresher training received and knowledge of the 2014 IMCI guidelines. The knowledge questions were adapted from an IMCI computer-based training course evaluation delivered by USAID in Kenya (20); this was selected due to its short length covering different elements of IMCI knowledge, and the questions should not have been previously seen by participants in our setting.

Data was collected by trained study staff who visited each facility at a pre-arranged time to conduct the audit and surveys. The study staff consisted of clinical officers and monitoring and evaluation officers. The data was collected with support from facility in-charge, pharmacy and health management information staff and included visual inspection of drug stocks to check quantities and expiry dates, equipment functionality and closed questions. Visual inspection by the study staff member was conducted to reduce potential recall and social desirability biases. Data was entered into android tablets using Open Data Kit Collect, with in-built cleaning and skip-pattern rules to promote data quality. The survey was self-completed on an android tablet for healthcare workers who were familiar with smart phone technology. If unfamiliar, the survey was interviewer administered.

Qualitative Data Collection

FGDs were held at healthcare facilities and led by a male Malawian researcher (AD) with experience of qualitative research, supported by a clinical officer to provide IMCI specific knowledge. Discussions were open to be conducted in Chichewa or English, depending on the content and preference of participants. Interviews and discussions were audio-recorded, transcribed and where necessary translated into English. Participants were reimbursed for their travel expenses and provided with refreshments.

Analysis

The audit data was described using proportions, and compared between facility types and districts using Chi-squared tests. Healthcare worker knowledge was summarised into a score with a

Confidential: For Review Only

maximum of 10 points. Mean scores were compared for the following independent variables: IMCI training received, years since training, refresher training received, years since refresher training, cadre, facility type and district. Multivariable linear regression analysis was conducted to assess the association between IMCI knowledge scores and training. The following confounders were considered: district (given different funding levels), facility type, health worker qualification and refresher course. Quantitative analysis was performed using Stata IC 16.0.

FGDs were analysed using a pragmatic framework approach, with pre-defined themes based on the topic guide (21) (Appendix 2). Emergent themes were coded during the analysis. Discrepancies were agreed through discussion, and the interpretation and conclusions shared and discussed with the wider study team. Coding was done by HH, with a sub-set double coded by CK. These codes were then discussed, refined and organised into themes, which were checked by AD for consistency with his interpretation and the local context.

Patient and Public Involvement

Patients and public were not involved in the design or execution of the study. Prior to starting, the protocol was presented to both District Health Management and District Executive Committees (which include local government and civil society representation) in both Zomba and Mchinji. Minor edits to the study plan were instituted following feedback and questions raised in these meetings.

Results

The total 47 health facilities were audited, with 16 in Mchinji and 31 in Zomba (**Table 1**), and 44% (n=531/1197) of clinical staff employed across these facilities were surveyed. All six planned FGDs were completed, with three in each district.

Facility Audits

Availability of seven IMCI drugs was assessed (**Table 2**). Lumefantrine-Artemether and cotrimoxazole were the most available (98% and 96%); while amoxicillin tablets and salbutamol nebuliser solution were the least available (28% and 36%). When available, the majority of drugs were found to be in date. Respiratory rate timers were the least available piece of equipment, with only 8 (17%) facilities across both districts having a functional device. This was followed by pulse oximeters (30%) and micro-nebulisers (32%, **Table 3**). MUAC tapes, scales and mRDTs were almost universally available. Health centres had the highest proportion of non-functional equipment.

Healthcare worker survey

Confidential: For Review Only

Overall 42% (n=222/531) of the survey respondents reported having IMCI training, with no difference by district (p-value=0.900). Of these, 38% had also received refresher training. The most common group to report training were HSAs (78%), while clinical officers, medical assistants and nurses all reported similar levels (45%), and only one attendant reported training. Training was most frequently received from NGOs (50%) followed by Ministry of Health (36%) and during qualification training (14%); refresher trainings were delivered by NGOs (64%) and Ministry of Health (36%). The median time since initial training was 7 years (95% confidence interval: 6, 8; range: 0 - 21).

The mean knowledge score, of a possible 10 points, was 3.96 (95% confidence interval: 3.83, 4.08; range: 0 – 8), with 65% scoring less than 5 (Web Appendix 3). Questions on referral decision using clinical scenarios had both the most correct responses (84% - severe dehydration with another sign of severity), and most incorrect (13% - severe dehydration without any other urgent signs). The multivariable regression found a statistically significant association between IMCI knowledge score and having received refresher training (coeff: 0.42; 95% CI: 0.01-0.82) - **Table 4**. However, training alone was not significantly associated with IMCI knowledge. Being a HSA or hospital attendant were both associated with a lower score compared to clinical officers (coeff: -0.82; 95% CI: -1.50, -0.14 and coeff: -1.61; 95% CI: -2.29, -0.92). Amongst those with training, there was a weak but statistically significant positive correlation between knowledge and years since training (correlation coeff: 0.213; p-value: 0.001).

Focus group discussions:

The qualitative data are presented under the following themes: IMCI implementation and practice, barriers to IMCI effectiveness, benefits of IMCI and sustainability.

1. IMCI implementation and practice

This theme includes healthcare workers understanding of IMCI, real-world adaptations, self-perceived quality of implementation, and training received. Overall IMCI was described as a system of assessment, a holistic approach to care for children and as a way to classify illness severity. Across the discussions it was apparent that there were inconsistencies in the implementation of IMCI, but groups generally described a process of weighing, initial clinical assessment, tests and then assigning a diagnosis. One group from Mchinji specifically talked of how a new electronic patient record system had resulted in more structure.

Healthcare workers discussed doing their best to deliver IMCI, in spite of challenges. Although also acknowledged that they do not always follow the guidelines, whether this was intentional was not clear:

Confidential: For Review Only

> "I guess some people are not using the IMCI approach so it is hard to assess properly. Because they are straight from school sometimes you need updates in order to know when everything is working properly and to give an alarm to the people who are using it" (Zomba, FGD 1)

In the groups, there was a mix between those who reported training and those who hadn't received any. Participants in one group described how staff had also received different types of training, depending on who delivered it:

> "There were some people who were trained by the Ministry of health, while others were retained by PSI [an international NGO…] it was a week-long training. But those who were trained by other organisations, the training lasted for three weeks." (Zomba, FGD 2)

2. Barriers to IMCI effectiveness

Three main barriers were discussed: community barriers, lack of adequate resources and lack of trained staff capacity. The issue of caregivers presenting late was a key example raised by several groups as a barrier to effective IMCI implementation. This resulted in tensions around communication and trust between caregivers and providers, and participants were aware of the compounding effects of high patient loads, stock-outs and not having 24-hour staffing.

> "When we ask her the time the child started getting sick she tells a lie by saying they started getting ill today. If we argue her response, you find that sometimes neighbours will confront her by saying the child started suffering sometime back only that she was busy running her business. That's the challenge we encounter sometimes." (Zomba, FDG 3)

While the presence of community structures to support the facilities were described, they noted that they did not always work together effectively. In terms of resources, this was raised as a major challenge for some, but not others who described well-functioning supply chain management for essential medications, such as antibiotics. Various scenarios of lacking guidelines, medications, diagnostics and equipment were given to highlight how these limit the ability to provide care for children

> "Sometimes you can find that the child has high fever and there is a need for the child to be given [anti-malarials], but before that you have to confirm with a malaria test, and if you don't have the test kit it becomes a big challenge"(Zomba, FDG 2)

Confidential: For Review Only

> "The guidelines are not there. Even if you are not trained you can use the guidelines to tell you what to do, but the guidelines are not there" (Zomba, FDG 1)

Multiple dimensions of staffing were raised, from high workloads restricting the amount of time that could be spent conducting an IMCI assessment, not all staff being trained, and lacking supportive supervision. One discussion with providers from a CHAM hospital however noted that they had no problems with staffing unless there were absences due to events such as funerals.

3. Benefits of IMCI

Benefits of IMCI included better clinical outcomes and better ways of working. Generally, many of the participants acknowledged that IMCI results in improved outcomes for children, and that this also translates into care and care-seeking practices amongst the community.

> "It helps mothers to have additional knowledge of caring [for their] children, through what she is advised at hospital by health workers" (Zomba, FDG 2)

IMCI was described as reducing the workload for hospitals, as frontline facility staff were able to treat conditions such as malaria. The structured and holistic approach was valued by participants in both districts, for example:

> "Yes, it provides a holistic approach to childhood illness, it gives a knowledge on how to treat under-five children. It is a lifesaving system." (Mchinji, FDG 1)

4. IMCI Sustainability

This theme addresses the long term sustainability of IMCI implementation in frontline facilities, with both end-user ownership and sustaining capacity discussed. Notably, this topic was only raised by healthcare providers in Mchinji and included both challenges and concrete recommendations. A key challenge was around the transfer of trained staff, especially when not all staff are sent for IMCI training. Around who attends training, there were concerns around transparency of who is sent for training, which can compound the issue.

> "Yes, it is a problem, for instance someone has gone for IMCI training and next month has been transferred, that means we will have a problem with IMCI because that person didn't finish sharing the information." (Mchinji, FDG 2)

Confidential: For Review Only

To promote sustainable programming there was a request for healthcare workers to be involved in the development process, and ensuring motivation and follow-up was sustained. NGO instituted programmes were specifically called out for this practice:

> "Another problem, it's you NGO people, you don't follow up the programmes" (Mchinji, FDG 3).

Confidential: For Review Only

Discussion

In this study we explored how IMCI is currently being implemented within two districts in Malawi. We found that there were mixed experiences with both implementation and access to essential supplies, notably with important differences between target infections. In terms of healthcare workers, we found refresher training to be more important in terms of IMCI knowledge than cadre of the healthcare worker and having training alone. These findings pose important topics for further exploration and discussion.

The ability to diagnose and treat respiratory conditions in general, and particularly pneumonia, in frontline facilities was poor. This is especially notable when compared to the almost universal access to malaria RDTs and treatment. ORS, as a treatment for diarrhoeal diseases, was also not readily available at government facilities. The IMCI guidelines recommend seven essential drugs for treating malaria, pneumonia, diarrhoea and ear infections. Several facilities lacked the necessary equipment and first-line treatments to diagnose and treat pneumonia, with the exception of cotrimoxazole, which is a first-line treatment for respiratory infections in children with HIV.

Donor funding for child health has steadily increased since the MDGs were introduced in 2000 and this has coincided with increased policies for malaria prevention and treatment in Malawi (10). This is reflected in well-funded malaria programmes in Malawi (Malawi Malaria Communication Strategy 2015-2020 (22)), and in the global funding landscape where malaria has been prioritised through the Global Fund, Bill and Melinda Gates Foundation and USAID (23, 24). This contrasts to pneumonia and diarrhoea, which despite having high burdens, do not receive the same level of strategic funding by large-scale donors. In particular, pneumonia has generally received lower research funding comparative to its global mortality burden (25). This vertical programming focus is often disease specific and detracts from the holistic approach to assessing childhood illness, which IMCI promotes. Further exploration of factors which promote sustainability at different levels of the health system could reveal important insights for future programming.

Resource availability impacts healthcare workers' abilities to adequately perform IMCI assessments and treat according to guidelines. This was apparent from the FGDs with providers, and consistent with previous studies from similar settings, which found better health worker performance in facilities with availability of all essential IMCI equipment (26). Given that some health facilities were better resourced than others, it would be useful to understand different funding allocation, stock management and personnel structures, to ascertain best practices.

The assessment of health care worker IMCI knowledge found more than half of healthcare workers scored under 50%, and those with higher qualifications did not necessarily score better. Completion of both IMCI training and a refresher course was associated with higher IMCI knowledge. This is in line with other studies on IMCI performance, where healthcare workers who had received IMCI training without a refresher course, performed similarly to those without training (27). It is important to note that while training doesn't necessarily translate into knowledge, knowledge does not always result in adherence to IMCI guidelines and improved practice. A previous study found that those with more experience felt that following the guidelines was not necessary (27). Time constraints and a high work load have also been reported to reduce adherence to IMCI guidelines (28).

We found a weak positive relationship between time since training and knowledge, which may reflect the importance of long-term clinical experience rather than training alone. A systematic review by Rowe et al. (2012) reported that healthcare workers performed better with IMCI training than without, but amongst those trained, over a third of ill children were not treated according to IMCI guidelines (29). Traditional training programs do not necessarily translate to adequate knowledge and skills without ongoing support, and findings from five sub-Saharan African countries found coaching and mentorship improved IMCI service delivery (30). The WHO recommends following up four weeks after the initial IMCI training course to help reinforce the skills learned, and continued supervision (31). There are examples of successful mentorship within child health (32, 33), and community case management in Malawi. However, as lacking supervision and mentorship was raised by healthcare workers in the FGDs, work is needed on effective adoption at frontline facilities.

The qualitative data highlighted a key issue around programme sustainability, and the need for local ownership. Training and refresher courses were often provided by NGOs (through external funding), not provided to all staff, and the decision for who is trained was not thought to be transparent. Given the frequent transfer of healthcare workers across different facilities, this results in an unequal distribution of workers with IMCI knowledge. It is therefore important that sustainability

https://mc.manuscriptcentral.com/bmjpo

Confidential: For Review Only

and handover plans are established by NGOs and research groups, which are agreed up-front with District Health Management Teams.

This study had three key limitations, firstly, that the two districts were selected purposively, and our findings may not be representative to other districts. For example, Mchinji has had multiple research projects in the last decade which have included IMCI refresher trainings and health system strengthening (e.g., McCollum et al. 2016 (34)). In particular, we did not include any district from the northern region. Secondly, the healthcare workers in the survey were those present on the day, and therefore may not cover the full range of providers (e.g., those who predominantly focus on outreach services). The frequency of healthcare worker cadres generally reflected the health workforce composition in Malawi, where HSAs constitute one third of the workforce and few clinical officers (19). The higher proportion of workers from Zomba is also expected given it has a larger population compared to Mchinji, and has traditionally received higher healthcare funding (17). Finally, assessment of practice was not performed, with direct observations of health worker performance posing several ethical and methodological challenges (e.g. the Hawthorne effect (35)). We therefore cannot comment on quality of IMCI care provision.

Malawi's early adoption and scaled-up implementation of IMCI has likely played a key role in the successes made in reducing under-five mortality, but further progress is needed to reach the SDG 3.2 goal. While we found expected challenges in staffing and resources, there are several opportunities for improvement and further research. Firstly, determining which combination of supervision, mentorship and re-training is most cost-effective within the specific context of frontline facilities, to ensure improved knowledge and subsequent quality of care. Secondly, to explore locally adapted IMCI best practices from those facilities which had good supply chain management, positive attitudes to staff transfers and sufficient trained staff capacity. Finally, supporting programming to move away from vertical financing with short term support, to the more holistic approach which IMCI promotes and considers strategies for embedded sustainability.

Confidential: For Review Only

Acknowledgements

We would like to thank the District Health Office in Mchinji and Zomba for supporting this project, and all the healthcare providers, facility managers, technicians and support start for sharing their time and knowledge. We acknowledge Christopher Kampinga, Tarcizious Phiri, Harry Liyaya (PACHI, Malawi) and Clifford Katumbi (College of Medicine, Malawi) for collecting the audit and survey data.

Funding

This study was funded by a project grant the Swedish Research Council 2017-05579. The funder did not play any role in the design, analysis or interpretation of the data. The analysis and write up was undertaken as part of an MSc student project (the student received a Global Grant Scholarship through Rotary International to undertake a graduate study program).

Conflict of Interest

None declared.

Author Contributions

The study was conceived by HH and CK and study protocols and data collection tools designed together with BZ, CM, LB, ND and AD. Quantitative data collection was overseen by BZ, LB and CM and qualitative data collection was conducted by AD, with support from BZ. Quantitative analysis was conducted by KK with input from CK and HH. Qualitative analysis was conducted by CK and HH, with input from AD and KK. The manuscript was drafted by KK, with significant contributions from CK, HH and AD. All authors read and approved the final manuscript.

Confidential: For Review Only

References

1. The United Nations Children's Fund WHO, World Bank Group, United Nations. Levels and Trends in Child Mortality. Report 2019. New York: UNICEF; 2019.
2. World Health Organisation. Integrated Management of Childhood Illness global survey report. Geneva: World Health Organisation; 2017.
3. GBD 2019 Diseases and Injuries Collaborators. Global burden of 369 diseases and injuries, 1990–2019: a systematic analysis for the Global Burden of Disease Study 2019. The Lancet. 2020.
4. World Health Organisation. Integrated Management of Childhood Illness (IMCI) 2019 [Available from: https://www.who.int/maternal_child_adolescent/topics/child/imci/en/.
5. World Health Organisation. IMIC: Improving Child Health. Geneva: WHO; 1999.
6. Jacobs M, Merson M. Introductory commentary: a strategic review of options for building on lessons learnt from IMCI and iCCM. British Medical Journal. 2018;362.
7. Boschi-Pinto C, Labadie G, Ramachandandran Dilip T, Oliphant N, Dalgish S, Aboubaker S, et al. Global implementation survey of Integrated Management of Childhood Illness (IMCI): 20 years on. BMJ Open. 2018;8.
8. Goga AE, Muhe LM, Forsyth K, Chopra M, Aboubaker S, Martines J, et al. Results of a multi-country exploratory survey of approaches and methods for IMCI case management training. Health Research Policy and Systems. 2009;7(1):18.
9. Nguyen DTK, Leung KK, McIntyre L, Ghali WA, Sauve R. Does Integrated Management of Childhood Illness (IMCI) Training Improve the Skills of Health Workers? A Systematic Review and Meta-Analysis. PLOS ONE. 2013;8(6):e66030.
10. Kanyuka M, Ndawala J, Mleme T, Chisesa L, Makwemba M, Amouzou A, et al. Malawi and Millennium Development Goal 4: a Countdown to 2015 country case study. The Lancet Global Health. 2016;4:e201-14.
11. Molyneux M, Molyneux E. Reaching Millennium Development 4. The Lancet Global Health. 2016;4(3):PE146-E7.
12. Wemos Foundation. Country Report Malawi. Minding the gap: Who is paying the health workers? Mind the funding gap:. Amsterdam; 2018.
13. Johansson E, Humphreys N, Carvajal-Aquirre L, Amouzou A, Hildenwall H. Determinants of Integrated Management of Childhood Illness (IMCI) non-severe pneumonia classification and care in Malawi health Facilities: Analysis of a national facility census,. Journal of Global Health. 2017;7(2).
14. Bjornstad E, Preidis G, Lufesi N, Olson D, Kamthunzi P, Hosseinipour M, et al. Determining the quality of IMCI pneumonia care in Malawian children. Paediatric International Child Health. 2014;34(1):29-36.
15. National Statistical Office. Malawi Population and Housing Census. Government of Malawi; 2019.
16. National Statistical Office/Malawi, ICF. Malawi Demographic and Health Survey 2015-16. Zomba, Malawi: National Statistical Office and ICF; 2017.
17. Borghi J, Munthali S, Million L, Martinez-Alvarez M. Health financing at district level in Malawi: an analysis of the distribution of funds at two points in time. Health Policy and Planning. 2018;33(1):59-69.
18. Makwero M. Delivery of primary health care in Malawi. African Journal of Primary Health Care & Family Medicine. 2018;10(1).
19. Angwenyi VA, C. Kondowe, K., Zulu Mutchiyeni JK, MCriel, B. Lazarus, J.V., Quinlan T, Bunders-Aelen J. Moving to a strong(er) community health system: analysing the role of community health volunteers in the new national community health strategy in Malawi. BMJ Global Health. 2018;3:1-10.
20. Quality Assurance Project. Evaluation of an IMCI Computer-based Training Course in Kenya. *Operations Research Results.* . Bethesda, MD: U.S. Agency for International Development (USAID) 2006.

Confidential: For Review Only

21. Gale NK, et al. Using the framework method for the analysis of qualitative data in multi-disciplinary health research. BMC Medical Research Methodology. 2013;13(1):117.
22. Ministry of Health Malawi. Malawi Malaria Communication Strategy 2015-2020. 2015.
23. USAID. President's Malaria Initiative Malawi Abbreviated Malaria Operational Plan FY 2019.
24. The Global Fund. Results Report. Geneva, Switzerland; 2019.
25. Head M, Fitchett J, Nageshwaran V, Kumari N, Hayward A, Atun R. Research Investments in Global Health: A Systematic Analysis of UK Infectious Disease Research Funding and Global health Metrics, 1997-2013. EBioMedicine. 2016;3:180-90.
26. Pariyo G, Gouws E, Bryce J, Burnham G, Uganda IMCI impact study team. Improving facility-based care for sick children in Uganda: training is not enough. Health Policy and Planning. 2005;20.
27. World Health Organisation. Global Strategy on Human Resources for Health: Workforce 2030 Geneva, Switzerland; 2016.
28. Lange S, Mwisongo A, Mæstad O. Why don't clinicians adhere more consistently to guidelines for the Integrated Management of Childhood Illness (IMCI)? Social Science and Medicine. 2014;104:56-63.
29. Rowe A, Rowe S, Holloway K, Ivanovska V, Mube L, Lambrechts T. Does shortening the training on Integrated
Management of Childhood Illness guidelines
reduce its effectiveness? A systematic review. Health Policy and Planning. 2012;27:179-93.
30. Manzi A, Hirschhorn L, Sherr K, Chirwa C, Baynes C, Awoonor-Williams J, et al. Mentorship and coaching support strengthening healthcare systems: lessons learned across the five Population Health Implementation and Training partnership projects in sub-Saharan Africa. BMC Health Services Research. 2017;17.
31. World Health Organisation. Guidelines for follow-up after training in the WHO/UNICEF course on Integrated Management of Childhood Illness for first-level health workers. 1999.
32. Asibon A, Lufesi N, Choudhury A, Olvera S, Molyneux E, Oden M, et al. Using a peer mentorship approach improved the use of neonatal continuous positive airway pressure and related outcomes in Malawi. Acta Paediatrica. 2020;109(4):705-10.
33. Guenther T, Mopiwa G, Nsona H, Qazi S, Makuluni R, Fundani CB, et al. Feasibility of implementing the World Health Organization case management guideline for possible serious bacterial infection among young infants in Ntcheu district, Malawi. PLOS ONE. 2020;15(4):e0229248.
34. McCollum ED, King C, Deula R, Zadutsa B, Mankhambo L, Nambiar B, et al. Pulse oximetry for children with pneumonia treated as outpatients in rural Malawi. Bull World Health Organ. 2016;94(12):893-902.
35. McCambridge J, Witton J, Elbourne D. Systematic review of the Hawthorne effect: New concepts are needed to study research participation effects. Journal of Clinical Epidemiology. 2014;67(3):267-77.

Confidential: For Review Only

Table 1. Health facility and healthcare worker cadre inclusion, by district

**Facilities**	**Total**	**Mchinji**	**Zomba**
Dispensary	7	4	3
Health centre	32	8	24
Rural hospital	3	1	2
CHAM hospital	5	3	2
Total	47	16	31
**Healthcare worker survey respondents**			
Clinical officer	17 (3%)	6 (3%)	11 (3%)
Medical assistant	47 (9%)	15 (8%)	32 (9%)
Nurse/midwife	105 (20%)	32 (17%)	73 (22%)
HSA	190 (36%)	69 (36%)	121 (36%)
Hospital attendant	172 (32%)	71 (37%)	101 (30%)
Total	531	193	338

Confidential: For Review Only

Table 2. *Drug availability and proportion of drugs in date by facility type*

	CHAM hospital (n=5)	Rural hospital (n=3)	Health Centre (n=32)	Dispensary (n=7)
Cotrimoxazole tablet	4 (80%)	3 (100%)	32 (100%)	6 (86%)
Amoxicillin tablets*	5 (100%)	1 (33%)	6 (19%)	1 (14%)
Amoxicillin syrup	4 (80%)	0	20 (63%)	4 (57%)
Lumefantrine-Artemether	5 (100%)	3 (100%)	32 (100%)	6 (86%)
Rectal artesunate	1 (20%)	1 (33%)	19 (59%)	1 (14%)
Oral rehydration salts	5 (100%)	0	16 (50%)	3 (43%)
Salbutamol tablets	5 (100%)	2 (67%)	15 (47%)	3 (43%)
Salbutamol nebuliser solution	4 (80%)	1 (33%)	5 (16%)	7 (100%)
Aminophylline injection**	2 (67%)	1 (100%)	3 (38%)	3 (75%)

**Availability of amoxicillin syrup and/or tables: 5 (100%) of CHAM facilities; 1 (33%) rural hospitals; 22 (69%) health centres; 4 (57%) dispensaries.*

*** Data only collected for Mchinji district (n=16)*

The following drugs were found not to be in date: 20% of ORS at CHAM facilities; 5% of amoxicillin syrup in health centres and 25% at dispensaries; 33% aminophylline at health centres.

Confidential: For Review Only

Table 3. Equipment Availability by facility type and proportion of functional equipment

	CHAM hospital (n=5)		Rural hospital (n=3)		Health Centre (n=32)		Dispensary (n=7)	
	**Available (n, %)**	**Functional (%)**	**Available (n, %)**	**Functional (%)**	**Available (n, %)**	**Functional (%)**	**Available (n, %)**	**Functional (%)**
Thermometer	5 (100%)	100%	3 (100%)	100%	25 (78%)	96%	7 (100%)	71%
Respiratory rate timer	1 (20%)	100%	0	-	6 (19%)	67%	2	100%
MUAC tape	5 (100%)	100%	3 (100%)	100%	32 (100%)	100%	7 (100%)	100%
Weighing scale	5 (100%)	100%	3 (100%)	100%	32 (100%)	100%	7 (100%)	100%
Micro-nebulizer	3 (60%)	100%	1 (33%)	100%	9 (28%)	78%	2	100%
Malaria RDTs	5 (100%)	100%	3 (100%)	100%	31 (97%)	100%	7 (100%)	100%
Pulse oximeter*	3 (60%)	100%	1 (33%)	100%	10 (31%)	70%	0	-

RDT = rapid diagnostic test; MUAC = mid-upper arm circumference

*Pulse oximetry is recommended but not essential according to IMCI guidelines

Confidential: For Review Only

Table 4. IMCI knowledge score description and multivariable linear regression analysis

**VARIABLES**	**Mean score (n=531)**				**Coefficient (95% CI)**	**p-value**
	N	Mean	Mchinji (n=193)	Zomba (n=338)		
Training						
No training	309	3.69	3.54	3.77	*Ref*	
IMCI training only	137	4.31	4.40	4.26	0.083 (-0.238, 0.405)	0.611
IMCI training + refresher	85	4.40	4.26	4.50	0.417 (0.012, 0.821)	0.043
Cadre						
Clinical Officer	17	4.76	4.67	4.82	*Ref*	
assistant Medical	47	4.57	4.73	4.50	-0.241 (-0.985, 0.503)	0.526
Nurse/midwife	105	4.62	4.63	4.62	-0.149 (-0.834, 0.536)	0.669
HSA	190	4.13	4.11	4.13	-0.820 (-1.500, -0.142)	0.018
Hospital Attendant	172	3.13	3.04	3.19	-1.605 (-2.286, -0.923)	<0.001
Facility type						
Dispensary	40	3.98	4.29	3.81	*Ref*	
Health Centre	378	4.01	3.91	4.04	0.136 (-0.298, 0.571)	0.538
Rural Hospital	113	3.80	3.76	3.92	-0.004 (-0.501, 0.493)	0.987
District						
Mchinji	193	3.87			*Ref*	
Zomba	338	4.01			0.001 (-0.264, 0.266)	0.993

Adjusted R-squared: 0.173

Confidential: For Review Only

Appendix 1: Summary of healthcare worker cadres and their role

**Qualification**	**Definition**
Health Surveillance Assistants	Community level workers with 6 weeks of initial health pre-service training. Generally, provide health promotion and preventive health care through door to door or outreach clinics. Diagnose and treat some illness as part of iCCM in more remote/underserviced areas [1].
Hospital Attendant	Workers who perform routine patient personal care as directed by more senior health personnel. It includes orderlies and nursing aides [2].
Medical Assistants	Two years of clinical training [1] to gain a certificate in clinical medicine. Perform clinical duties (except for surgical procedures) [3].
Clinical Officer	Mid-level practitioners who receive 3-4 years of training and often manage health facilities and fill the role of doctors due to workforce shortages [1, 4]

1. Makwero M. Delivery of primary health care in Malawi. *African Journal of Primary Health Care & Family Medicine*. 2018;10(1)
2. Malawi Health Workforce Observatory. *Human Resources for Health Country Profile Malawi,*. 2010.
3. Muula A. Case for Clinical Officers and Medical Assistants in Malawi. *Croatian Medical Journal*. 2009;50(1):77-78.
4. Jiskoot P. On-the-job training of clinical officers in Malawi. *Malawi Medical Journal*. 2008;20(3):74-77.

Confidential: For Review Only

Appendix 2: Healthcare worker focus group discussion topic guide

1. Current IMCI

- When did you last have IMCI training? Can you describe the training?
- Can you describe what IMCI is?
- Do you think you implement IMCI in your setting?
- Are there any barriers you face in implementing IMCI?
- Are there any benefits you think of implementing IMCI?

2. Current emergency treatment

- Can you give a recent example of child you saw, that you would describe as an emergency?
    - Probe: do others recognise this example, ask for multiple different ones, you can give an example
- Can you describe what you did with this child?
    - Probe: would everyone do the same, what would they do different
- What would you have liked to do in this situation?
- How did you feel about your management of this case?
- How often do you see children like this?
- Do you use any tools or algorithms (guidelines) when assessing emergency cases?
- When managing emergency cases, how do you work? Is it alone, or in a team? Who is part of the team?

3. Current referral procedure

- How often do you refer a child to the hospital? For example, this week how many children has everyone referred?
- Can you describe the process of referral?
    - Probe: documentation, explaining to the caregiver, organising transport
- Do all caregivers complete the referral?
- Are there any barriers you face in referring children?
- Are there any benefits to referring children?

4. ETAT awareness

- How do you decide which order to see patients in your clinic?
- Have you heard of ETAT? Can you describe ETAT?
- Have you ever had training in ETAT? What about other types of triaging?

5. ETAT preparedness

- What steps would need to happen for you implement ETAT?
- Do you have the equipment you need? Can you give examples?
- Do you have the staff you need?
- Are there any barriers to implementing ETAT? And barriers to adequate management of severely ill children?
- Are there any benefits to implementing ETAT in your setting?

Confidential: For Review Only

Appendix 3: Summary of healthcare worker IMCI knowledge scores, with 10 being the maximum score

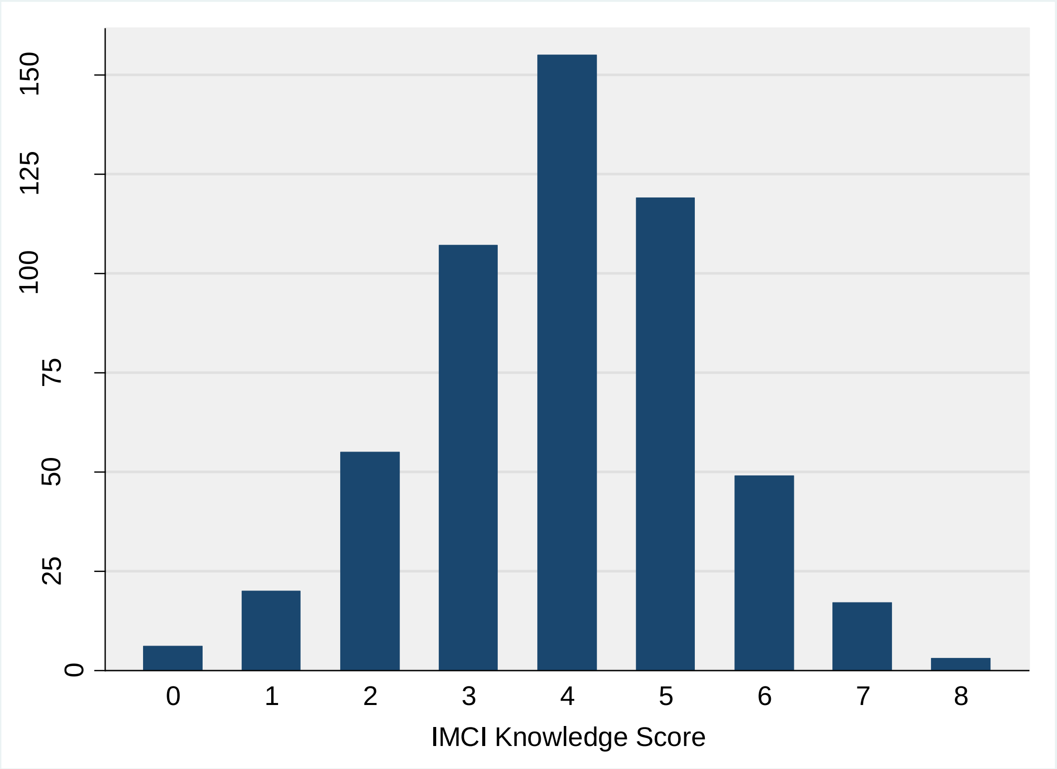

For Review Only

Appendix 4: Healthcare provider IMCI training and knowledge survey

1.1 Have you ever had IMCI training?	Yes / No	If YES, go to 1.4
1.2 Have you ever been given an orientation or briefing on IMCI?	Yes / No	If NO, go to 1.9
1.3 Who gave you this orientation/ briefing?	1. Facility in-charge<br>2. Other facility staff<br>3. DHMT representative<br>4. Other	Go to 1.9
1.4 When was your first training?	_______ year	
1.5 Who provided this first training?	1. MoH/DHMT<br>2. NGO<br>3. Research project<br>4. During school/training<br>5. Other	
1.6 Have you had a refresher training or active mentorship since?	Y / N	If NO, go to 1.9
1.7 When was the refresher?	_______ year	
1.8 Who provided this refresher training?	1. MoH / DHTM<br>2. NGO<br>3. Research project<br>4. During school/training<br>5. Other	Select all that apply
1.9 What is a child's classification if he is 10 months old, has had a cough that lasted two days, has a breathing rate of 46 breaths per minute and chest indrawing?[1]	1. Cough or cold<br>2. Pneumonia<br>3. Severe pneumonia<br>4. Very severe febrile disease<br>5. Don't know	Select one
1.10 What are the four main symptoms for which every sick child should be checked?	1. Malnutrition, cough, vitamin A, ear problems<br>2. Anemia, fever, diarrhea, ear problem<br>3. Cough, diarrhea, malnutrition, ear problem<br>4. Cough, diarrhea, fever, ear problem<br>5. Don't know	Select the best answer
1.11 For each of the following cases, select **Yes** if urgent referral is needed or select **No** if urgent referral is not needed.		
1.11a A 6-month-old boy does not have general danger signs. He is classified with: MASTOIDITIS and NO ANEMIA AND NOT VERY LOW WEIGHT	1. Yes<br>2. No<br>3. Don't know	
1.11b A 7-month-old girl does not have general danger signs. She is classified with: COUGH and DIARRHEA	1. Yes<br>2. No<br>3. Don't know	

Confidential: For Review Only

1.11c A 9-month-old boy is lethargic. He is classified with: DIARRHEA and WITH SEVERE DEHYDRATION	1. Yes<br>2. No<br>3. Don't know	
1.11d A 2-year-old girl does not have general danger signs. She is classified with: DIARRHEA and SEVERE DEHYDRATION and SEVERE MALNUTRITION	1. Yes<br>2. No<br>3. Don't know	
1.12 If a child has any of the four general danger signs, you should urgently refer him to hospital for treatment, These signs are:	1. Unable to drink/feed<br>2. Severe cough<br>3. Convulsions<br>4. Vomiting everything<br>5. Lethargy / unconsciousness<br>6. Bloody stools<br>7. Don't know	Select four from the list
1.13 According to IMCI, a mother of a sick child should be counselled about what topics:	1. Importance of fluids and feeding<br>2. When to return to the clinic immediately<br>3. Her own health<br>4. Immunisation<br>5. When to return for a follow-up visit<br>6. The treatments being given to the child<br>7. Family planning<br>8. Don't know	Select all that apply
1.14 What are two signs that are used to classify severe malnutrition	1. Small arm circumference<br>2. Visible severe wasting<br>3. Oedema of both feet<br>4. Severe dehydration<br>5. Don't know	Select two from the list
1.15 To classify the dehydration status of a child with diarrhoea you will look:	1. At the general condition<br>2. For sunken eyes<br>3. For oedema of both feet<br>4. If the child is drinking eagerly<br>5. For palmar pallor<br>6. For a swollen abdomen<br>7. Don't know	Select all that apply

Confidential: For Review Only
